# Adsorption and Thermal Stability of Phenylphosphonic Acid on Cerium Oxides

**DOI:** 10.1021/acs.jpcc.5c04065

**Published:** 2025-08-13

**Authors:** Viacheslav Kalinovych, Lesia Piliai, Yuliia Kosto, Sascha L. Mehl, Tomáš Skála, Kevin C. Prince, Iva Matolínová, Ye Xu, Nataliya Tsud

**Affiliations:** † Faculty of Mathematics and Physics, Department of Surface and Plasma Science, 37740 Charles University, V Holešovičkách 2, Prague 18000, Czech Republic; ‡ 18474 Elettra-Sincrotrone Trieste S.C.p.A., in Area Science Park, Strada Statale 14, km 163.5, Basovizza, Trieste 34149, Italy; § Cain Department of Chemical Engineering, 5779 Louisiana State University, Baton Rouge, Louisiana 70808, United States

## Abstract

This paper reports on a study of the adsorption and thermal stability of phenylphosphonic acid (PPA) adsorbed by physical vapor deposition on the surfaces of epitaxial cerium oxide films of different structure, stoichiometry and composition. Advanced analytical methods based on photoelectron spectroscopy combined with DFT calculations showed that the binding of PPA to cerium oxide is through the phosphonate group, while the decomposition temperature is defined by the nature of the oxide. Tridentate PPA species are present on all substrates (CeO_2_, CeO_1.7_, Ce_2_O_3_, and Ce_6_WO_12_), indicating a strong affinity of PPA for cerium oxide. The presence of vacancies in the oxide influences the molecular orientation. The phenyl ring of the PPA tilts about 10° more toward the surface plane of the reduced cerium oxides compared to CeO_2_, which is attributed to the adsorption of phosphonate groups on Ce^4+^ and Ce^3+^ cations. The PPA adlayer is more stable on the surfaces with higher concentrations of oxygen vacancies and/or Ce^3+^ cations, increasing the temperature to initiate cleavage of the P–C bond from 225 °C for PPA/CeO_2_ to 350 °C for the other systems. The PPA decomposition is signaled by the desorption of carbonaceous species above a critical temperature, while the phosphorus species remain stable even after annealing at 450 °C for all the cerium oxides. Overall, the results provide a comprehensive understanding of the binding of PPA to cerium oxides, allowing further development of functionalization strategies for inorganic materials by phosphonic acids.

## Graphical abstract

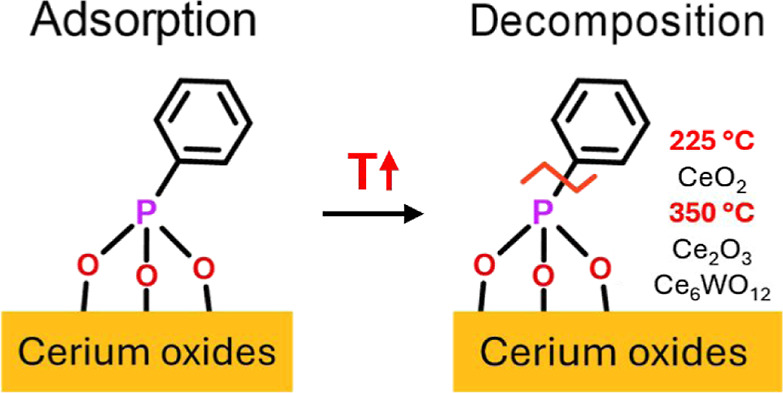

## Introduction

Hybrid materials, combining organic and inorganic substances, are a vital driver of technological advancements.^1,2^ The efficient design of hybrid materials requires an understanding not only of the properties of each substance, but also of the interface between them. The interface is particularly important when considering organic molecules bound to inorganic substances as hybrid materials, or as possible linkers in the formation of more complex composites.^3–5^ The interface is often modeled using simplified systems where the organic molecules are adsorbed onto thin films of the inorganic material.^6–14^ In this work, the molecular binding of phenylphosphonic acid (PPA; C_6_H_5_PO­(OH)_2_) to cerium oxide films was studied using experimental and theoretical model approach.

PPA consists of a phenyl ring and a phosphonate group linked by a P–C bond. Adsorption of phosphonic acids on an inorganic substrate through the −PO­(OH)_2_ group can lead to the formation of up to three bonds with the substrate, i.e. mono-, bi-, and tridentate binding modes. Model studies of the adsorption of phosphonic acids on metal oxides have shown that the molecular binding depends on the surface structure,^6–14^ the presence of oxygen vacancies (V_O_),^14,15^ and the molecular coverage.^9,11,12^

The surface structure of the oxide determines the concentration of adsorption sites, their arrangement and accessibility to the phosphonate groups. For example, the influence of the surface structure has been experimentally demonstrated for low coverage deuterated PPA (DPPA, C_6_H_5_PO­(OD)_2_) adsorbed on epitaxial thin films of CoO(100), CoO(111), and Co_3_O_4_(111).^11,12^ On the CoO(100) surface, which has the highest concentration of Co^2+^ sites, DPPA adsorbed on three Co^2+^ cations resulting in a bridging tridentate binding via the phosphonic group.^12^ On Co_3_O_4_(111), DPPA adsorbed on only one Co^2+^ cation in a chelating tridentate geometry via the P–O bonding due to the low concentration of Co^2+^ cations on the surface.^11^ Finally, on CoO(111), the O-terminated surface partially covers the underlying Co^2+^ cations, making them less accessible to the phosphonate groups and limiting DPPA adsorption to the bridging bidentate configuration, i.e. to more than one cobalt cation.^12^ DFT studies of the adsorption of phosphonic acid (HPO­(OH)_2_) on rutile TiO_2_(110)^7^ and anatase TiO_2_(101)^6,7^ predicted that the molecular bonding is limited to only mono- and bidentate modes due to the low concentration of 5-fold coordinated Ti adsorption sites. The tridentate adsorption complexes were found to be unstable due to the geometric constraints of the third P–O–Ti bond.^6,7^ The findings were later confirmed experimentally, where the mono- and/or bidentate binding modes of various phosphonic acids were observed on both TiO_2_(110)^8,13,14^ and TiO_2_(101).^9,10^

The presence of oxygen vacancies provides additional adsorption sites for molecules. This has been demonstrated in the DFT study of PPA adsorption on clean and oxygen-deficient WO_3_(001) surfaces.^15^ Due to the low concentration of 5-fold coordinated W sites, only the monodentate binding mode of PPA was found to be stable on the clean surface.^15^ In contrast, on an oxygen-deficient WO_3_(001) surface, oxygen vacancies were filled by O from the phosphonate group, resulting in a stable bidentate configuration of PPA with stronger adsorption compared to the clean surface.^15^ The influence of oxygen vacancies on molecular binding was also observed experimentally during the annealing of submonolayer PPA on rutile TiO_2_(110).^14^ Initially, PPA molecules adsorbed in a bridging bidentate configuration. Annealing at 225 °C created vacancies due to water desorption, which were subsequently filled by the formation of Ti–O–P bonds, changing the PPA bonding to a mixed bridging bi/tridentate.^14^

Another parameter affecting phosphonic acid bonding is the availability of adsorption sites. As the monolayer (ML) approaches completion, i.e. more and more adsorption sites are filled, fewer bonds are formed for each adsorbed molecule, mainly due to geometric constraints. This has been observed, for example, for different coverages of PPA on anatase TiO_2_(101)^9^ and DPPA on Co_3_O_4_(111).^11^ In particular, for the PPA/TiO_2_(101) system, approaching the saturation coverage resulted in a change in the adsorption geometry from bridging bidentate to a mixture of mono/bidentate configurations.^9^ In the case of DPPA on Co_3_O_4_(111), the binding was chelating tridentate at low coverage and chelating bidentate at high coverage.^11^ In contrast, a transition from bridging bidentate to bridging tridentate binding of DPPA on CoO(111) was observed with increasing coverage due to surface reconstruction.^12^

In addition to identifying the adsorption geometry and coverage, the thermal stability of the molecular layers is often probed by annealing of model systems.^12–14,16,17^ Thermal treatment has been shown to alter the bonding between the phosphonic acid adlayer and the substrate^12–14^ or induce decomposition.^16,17^ Annealing can also trigger the desorption of surface hydroxyl groups from the oxide surface^13^ and/or the formation of vacancies,^14^ which provide additional adsorption sites for the molecules. When the critical temperature is reached, the decomposition is typically initiated by P–C bond scission between the phosphonate group and the organic moiety^17^ or by bond-breaking within the organic group.^16^ In general, the phosphonic acids have demonstrated high thermal stability on metal oxides with decomposition starting above 250 °C,^14,16,17^ making them promising ligand molecules for the functionalization of inorganic materials.

Among metal oxides, cerium oxide has unique properties due to its ability to undergo a shift in oxidation state from 4+ to 3+ in response to redox interactions or oxygen exchange with the environment. As a result, it exhibits high catalytic activity and is used to remove air pollutants,^18^ reform hydrocarbons,^19^ produce hydrogen,^20^ etc. It is well-known that the structure, composition and stoichiometry of cerium oxide can affect its surface reactivity.^21–23^ The interaction of phosphonic acids with the surface of cerium oxide has not been extensively investigated in the literature. To our knowledge, only one study has reported the reactivity of a phosphonic acid, namely dimethyl methylphosphonate (DMMP, CH_3_PO­(OCH_3_)_2_), on model CeO_2_(111) and CeO_*x*_(111) films.^24^ The adsorption of DMMP occurs via its phosphonate group, which undergoes demethylation upon annealing, resulting in a tridentate bonding to the substrate at and above 225 °C. Furthermore, the adsorption behavior of DMMP on both oxide films was found to be the same and independent of the surface stoichiometry.^24^ In addition, several DFT studies have investigated the adsorption of several phosphates on CeO_2_(111).^25,26^ The calculations indicate that phosphates adsorb via the PO_2_(OH)_2_ functional group, which undergoes complete deprotonation upon adsorption, favoring the bridging tridentate bonding. It was also shown that the presence of a surface oxygen vacancy next to the adsorbed functional group stabilized the phosphate adsorption.^25^

In order to obtain a comprehensive description of PPA binding to cerium oxide as a function of surface structure, stoichiometry and composition, the following well-defined films were prepared: fully oxidized CeO_2_(111)/Cu­(111), partially reduced CeO_1.7_/Cu­(111), fully reduced Ce_2_O_3_(111)/Cu­(111), and mixed Ce_6_WO_12_(100)/WO/W­(110). CeO_2_ and Ce_2_O_3_ are fully stoichiometric, i.e. extreme forms with only Ce^4+^ and Ce^3+^ cations, respectively; CeO_1.7_ represents a mixture of both, which is a common case modeling a realistic intermediate situation, e.g. when CeO_2_ is partially reduced due to lack of surrounding oxygen and/or while catalyzing a chemical reaction; Ce_6_WO_12_ is then a ternary oxide with pure Ce^3+^ and W^6+^ cations. The advantage is that all of these oxides can be easily prepared in situ as clean compounds with a well-defined structure^27,28^ and without impurities. The binding properties and adsorption of PPA on these model systems were studied by synchrotron radiation photoelectron spectroscopy (SRPES), resonant photoelectron spectroscopy (RPES) and X-ray photoelectron spectroscopy (XPS) under ultrahigh vacuum (UHV) conditions. The spatial orientation of the PPA adlayer was analyzed using near-edge X-ray absorption fine structure (NEXAFS) spectroscopy. The thermal stability of the PPA molecular adlayer was investigated during thermal annealing from room temperature up to 450 °C. The experimental results were corroborated by DFT calculations.

## Methods

### Experimental Section

The experiments were performed at the Materials Science beamline, Elettra synchrotron (Trieste, Italy). The experimental setup consists of a Specs Phoibos 150 hemispherical electron energy analyzer, a dual-anode Mg/Al X-ray source, a Ce evaporator, low energy electron diffraction (LEED) optics and a sample manipulator with a K-type thermocouple. The base pressure in the analysis chamber was 3 × 10^–10^ mbar.

Phenylphosphonic acid powder (C_6_H_5_PO­(OH)_2_, 98% purity) was supplied by TCI Organic Chemistry and used without further purification. PPA deposition was carried out in the preparation chamber (the base pressure of 3 × 10^–9^ mbar) using a Knudsen cell evaporator. The alumina crucible filled with the PPA powder was degassed at 95 °C and then heated to 105 °C for deposition. The substrate was kept at room temperature during deposition, and the chamber pressure was 2 × 10^–8^ mbar.

The P 2p, W 4f, C 1s, and O 1s core level spectra were recorded at photon energies of 230, 230, 410, and 630 eV with total resolution of 0.28, 0.28, 0.40, and 0.63 eV, respectively. The valence band (VB) spectra were measured at 121.4, 124.8, and 115 eV with total resolution of 0.2 eV. The VB spectra obtained at 121.4 and 124.8 eV correspond to the resonance enhancement of the Ce^3+^ (emission from Ce 4f states located at a binding energy (BE) of about 1.4 eV) and Ce^4+^ (emission from hybridized oxygen–cerium states at about 4.0 eV) cations, denoted D­(Ce^3+^) and D­(Ce^4+^), respectively (for details see Figure S1 in the Supporting Information). The VB spectrum measured at 115 eV photon energy corresponds to the off-resonance ionization of the Ce^3+^ and Ce^4+^ states and is used as a reference. The resonance enhancement ratio (RER) was determined as D­(Ce^3+^)/D­(Ce^4+^) and provides information about the oxidation state of the cerium cations on the surface with high sensitivity.^29^ Al K_α_ radiation (1486.6 eV) was used to measure the P 2p, W 4f, C 1s, O 1s, Ce 3d, and Cu 2p_3/2_ core levels with total resolution of 1 eV. The emission angles were 0° and 20° with respect to the surface normal for SRPES and XPS, respectively. The spectra obtained with synchrotron radiation were normalized to the incident photon flux. The accuracy of the photon energy was checked by measuring the Fermi level of a clean Cu(111) surface. The effective thickness of the oxide films and molecular adlayers was calculated from the attenuation of the substrate photoemission signals. The molecular coverage was also estimated assuming a discontinuous molecular film on the oxide surface. Details of the effective thickness and coverage estimation are given in Section S2 of the Supporting Information.

NEXAFS spectra were measured at the C K-edge using the C KLL partial Auger yield at normal emission (30° angle between beam and surface, NE), grazing incidence (10°, GI), and normal incidence (90°, NI) of the photon beam with respect to the surface. The polarization of the light is assumed to be 80% linear. The spectra were normalized to the intensity of the Cu 3p peak taken from the clean Cu(111) surface in the same photon energy range. Further details of the NEXAFS data treatment are given in Section S3 of the Supporting Information.

The following substrates were used for PPA deposition:

#### Fully Oxidized CeO_2_(111) (2.0 nm Thick) on Cu(111)

Prior to the reactive cerium deposition in an oxygen atmosphere, the Cu(111) single crystal (8 mm diameter, 2 mm thickness, MaTecK, 99.999%) was cleaned by cycles of Ar^+^ ion sputtering (1 keV) followed by UHV annealing at 500 °C. After confirming the cleanliness of the substrate and the long-range order of the surface by checking the C 1s region and LEED, respectively, cerium (Goodfellow, 99.99%) was deposited on Cu(111) at 250 °C in O_2_ (5 × 10^–7^ mbar).^27^ After the deposition, the sample was annealed for a further 5 min in O_2_. The CeO_2_ film is characterized by a (1.5 × 1.5) LEED pattern (Figure S3 a of the Supporting Information) and consists of Ce^4+^ cations on the surface, as monitored by RPES.

#### Fully Reduced Ce_2_O_3_(111) (4.0 nm Thick) on Cu(111)

First, the CeO_2_(111)/Cu­(111) buffer was prepared as described above. Then, 1.5 nm of metallic cerium was evaporated in UHV while the substrate was kept at room temperature. To obtain a reduced oxide, the sample was then annealed in UHV at 600 °C. During this process, a homogeneous, fully reduced cerium 3+ oxide film is formed due to the diffusion of oxygen vacancies toward the buffer oxide.^27^ The film contains Ce^3+^ cations and a maximum number of the oxygen vacancies, resulting in a (4 × 4) LEED pattern (Figure S3b of the Supporting Information).

#### Partially Reduced CeO_1.7_ (3.0 nm Thick) on Cu(111)

The preparation procedure is similar to that for the fully reduced film, except that a smaller amount (1.0 nm) of metallic cerium was deposited on the buffer oxide film in UHV. As a result, both Ce^4+^ and Ce^3+^ cations and oxygen vacancies are present in the oxide, with a higher concentration of Ce^3+^ cations near the surface.^27^

#### Mixed Ce_6_WO_12_(100) (0.6 nm Thick) on W(110)

The W­(110) single crystal (10 mm diameter, 1.5 mm thickness, MaTecK, 99.999%) was cleaned by several cycles of Ar^+^ ion sputtering (1 keV) and annealing in UHV at 900 °C. The surface cleanliness was checked by C 1s core level and surface ordering by LEED. Prior to the cerium evaporation, the W(110) crystal was preoxidized to form a WO adlayer by heating in O_2_ (5 × 10^–7^ mbar) at 600 °C. The cerium was deposited on top of the WO film by evaporation in O_2_ (5 × 10^–7^ mbar) on the substrate held at 500 °C.^28^ The sample was then flash annealed in UHV at 900 °C. This procedure resulted in the formation of an epitaxial Ce_6_WO_12_(100) film with only Ce^3+^ cations. The LEED pattern of the oxide film is shown in Figure S3c of the Supporting Information.

### Computational

Periodic, spin-polarized DFT calculations were performed in the generalized gradient approximation (GGA-PBE)^30^ and with the optB86b van der Waals (vdW) functional^31^ as implemented in the Vienna Ab initio Simulation Package (VASP).^32^ The latter functional was used in anticipation of possible vdW contributions in the adsorption of an aromatic compound. Potentials due to core electrons were described using the projector-augmented wave (PAW) method,^33,34^ and valence states [Ce­(4f5s5p5d6s), O­(2s2p), P­(3s3p), C­(2s2p), H­(1s)] were expanded in a plane wave basis set with a cutoff energy of 400 eV.

The DFT+*U* approach of Dudarev et al.^35^ was employed with a *U*_eff_ value of 5 eV. At this *U*_eff_ value, the equilibrium lattice constant of bulk CeO_2_ was calculated to be 5.500 Å (GGA-PBE) and 5.427 Å (optB86b-vdW), in good agreement with the experimental value of 5.41 Å,^36^ while the equilibrium lattice constant of *c*-Ce_2_O_3_ (bixbyite) was calculated to be 11.27 Å (GGA-PBE) and 11.21 Å (optB86b-vdW) vs the experimental value of 11.16 Å.^37^ The thermodynamically most stable (111) facet of CeO_2_ was modeled as a slab consisting of three O–Ce–O trilayers with a (3 × 3) surface unit cell, and the thermodynamically most stable (111) facet of Ce_2_O_3_ was modeled as a slab consisting of four O–Ce–O trilayers with a (4 × 4) surface unit cell. Periodic slabs were separated by at least 13 Å of vacuum with dipole decoupling applied in the *z* direction.^38^ The surface Brillouin zone was sampled at the Γ point only. An isolated PPA molecule in the gas phase was optimized in an 18.0 × 18.1 × 18.2 Å^3^ simulation cell with dipole decoupling applied in all directions and the reciprocal space integration at the Γ point only.

Adsorption of PPA was modeled with one PPA molecule per surface unit cell, which corresponded to one per 9 Ce cites or a local coverage of 0.11 monolayer (ML) for CeO_2_(111), and one per 16 Ce sites or a local coverage of 0.06 ML for Ce_2_O_3_(111). The top trilayer in the CeO_2_(111) slab and top two trilayers in the Ce_2_O_3_(111) slab, plus any adsorbate thereon, were fully relaxed, while the rest of each slab was fixed at the respective bulk coordinates. Geometry optimization was carried out until the maximum residual force was ≤0.03 eV/Å in each relaxed degree of freedom. The adsorption energy, Δ*E*_ads_, was evaluated as Δ*E*_ads_ = *E*_total_ – *E*_slab_ – *E*_gas,_ where *E*_total_, *E*_slab_, and *E*_gas_ refer to the total energies of the slab with PPA adsorbed thereon, the clean slab, and PPA in the gas phase, respectively. A more negative value of Δ*E*_ads_ indicates stronger bonding between the adsorbate and CeO_2_(111). The GGA-PBE adsorption configurations were further reoptimized using the optB86b vdW functional to capture possible vdW contributions to adsorption. The reaction energy (Δ*E*_rxn_) is defined to be the difference between the final and the initial states of a reaction step, as Δ*E*_rxn_ = *E*_final_ – *E*_initial_.

## Results

The PPA molecules were deposited on CeO_2_, CeO_1.7_, Ce_2_O_3_, and Ce_6_WO_12_ for 5, 5, 5, and 23 min, respectively. The effective thickness and the coverage of the molecular adlayer on the surface were estimated using eqs S1 and S2 in the Supporting Information and summarized in Table 1. The effective thickness was calculated from the intensities of the XPS spectra obtained after molecular deposition and then after annealing at 75 °C, in order to exclude the influence of physisorbed molecules. Note that the effective thickness of the PPA adlayer is an estimate value within a continuum molecular film model and is only qualitative in nature. The attenuation of the Cu 2p_3/2_ (for CeO_2_, CeO_1.7_, and Ce_2_O_3_) and W 4f (for Ce_6_WO_12_) substrate signals was used for this purpose. Similar effective thickness values of 0.22, 0.18, and 0.17 nm were found for as deposited PPA adlayers on CeO_2_, CeO_1.7_, and Ce_2_O_3_ oxides, respectively, confirming the stable deposition rate. Annealing at 75 °C affected these values, increasing them to 0.27 nm for CeO_2_ and much higher to 0.35 nm for both CeO_1.7_ and Ce_2_O_3_, likely reflecting the change in adsorption geometry, as the photoemission signals show only minimal changes (see below). The highest effective thickness value of 0.72 nm was found for the Ce_6_WO_12_ oxide, which was consistent with a longer deposition time (23 min). In addition, annealing at 75 °C had little effect on this value.

**Table 1:** Calculated Values of Effective Thickness, Coverage, and Stoichiometry of PPA on CeO_2_, CeO_1.7_, Ce_2_O_3_, and Ce_6_WO_12_ Oxide Films before and after Annealing at 75 °C

system	effective thickness, (nm)	coverage, (ML)	stoichiometry, P:C ratio
PPA/CeO_2_(111)			
25 °C	0.22		1.15:6
75 °C	0.27	0.07	0.80:6
PPA/CeO_1.7_			
25 °C	0.18		1.00:6
75 °C	0.35	0.10	0.97:6
PPA/Ce_2_O_3_(111)			
25 °C	0.17		0.90:6
75 °C	0.35	0.08	0.80:6
PPA/Ce_6_WO_12_(100)			
25 °C	0.72		0.80:6
75 °C	0.74	0.15	1.10:6

The PPA molecular coverage (in monolayers, MLs) was estimated from the P 2p and Ce 3d XPS intensities after heating to 75 °C, and using eq S2 in the Supporting Information. Values below saturation coverage (estimated to be ∼0.3 ML based on the geometric relationship between the CeO_2_ lattice cell size and PPA molecular dimensions) were obtained for all systems: 0.07, 0.10, 0.08, and 0.15 ML for CeO_2_, CeO_1.7_, Ce_2_O_3_, and Ce_6_WO_12_ oxides, respectively. These values reflect well the effective thickness data.

The stoichiometry of the PPA adlayer, i.e. the intensity ratio P:C is also given in Table 1. Oxygen was not included in the stoichiometry estimation because it is impossible to clearly distinguish the PPA oxygen contribution to the O 1s signal. The values obtained for CeO_2_ and Ce_2_O_3_ after PPA deposition are very close to the expected P:C ratio of 1:6. After the first annealing, the relative intensity of the phosphorus signal decreases for both systems, indicating upright molecular adsorption via phosphonate oxygen atoms bonded to the surface and partial attenuation by the phenyl ring. This change was less pronounced for the CeO_1.7_ oxide. The opposite tendency was observed for the PPA/Ce_6_WO_12_ system: the ratio of 0.8:6 after the PPA deposition changed to 1.1:6 after 75 °C heating, again probably due to a slight change in the adsorption geometry.

### RPES

The evolution of the RER, D­(Ce^3+^)/D­(Ce^4+^), and the separate resonance enhancement signals, D­(Ce^3+^) and D­(Ce^4+^), for the CeO_2_ and CeO_1.7_ oxides before and after the PPA deposition and during the annealing are shown in parts a, b and d, e, respectively, of Figure 1. For the Ce_2_O_3_ (Figure 1f) and Ce_6_WO_12_ (Figure 1c) films, only the resonance enhancement D­(Ce^3+^) is plotted since the Ce^4+^ cations were not present on the clean oxide surface, i.e. no D­(Ce^4+^) were observed in RPES VB spectra.^27,39^

**Figure 1:**
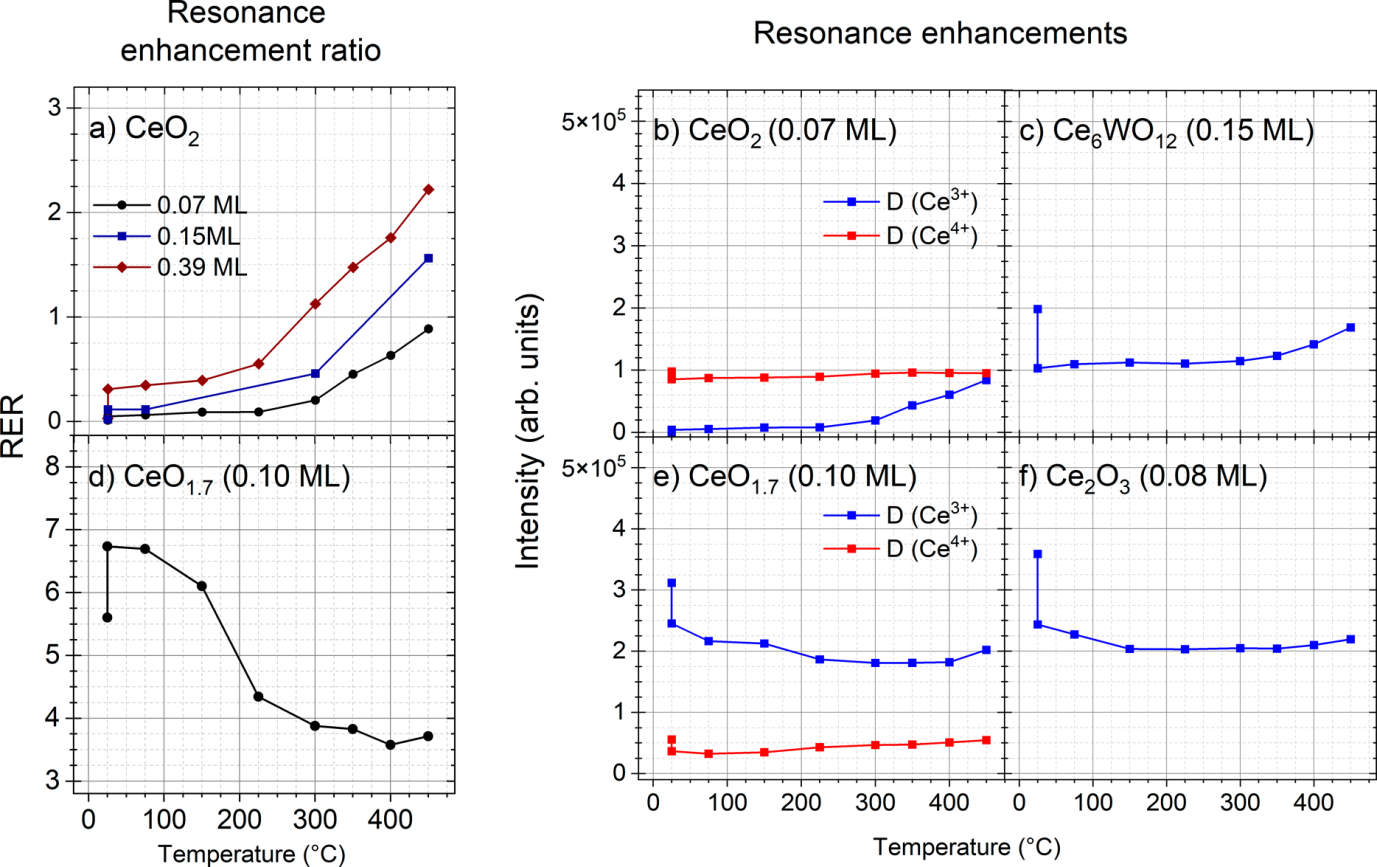
(a,d) The resonance enhancement ratio D­(Ce^3+^)/D­(Ce^4+^) and (b,c,e,f) the resonance enhancements D­(Ce^3+^) (blue) and D­(Ce^4+^) (red) after PPA deposition on (a,b) CeO_2_, (d,e) CeO_1.7_, (f) Ce_2_O_3_, (c) Ce_6_WO_12_ versus annealing temperature. The values before PPA deposition are plotted as the first point at 25 °C. In addition to the data for the 0.07 ML PPA/CeO_2_ system, the RER values for 0.15 and 0.39 ML PPA coverage are also shown in part a of the figure.

After 0.07 ML PPA deposition on CeO_2_(111), an increase of RER from 0 to 0.05 was observed, indicating a slight reduction of the oxide surface (Figure 1a, black). Then, with increasing temperature, the RER value gradually increased to 0.09. Above 225 °C the slope of the curve changed drastically. The final treatment at 450 °C gave the highest value of 0.9. It is worth mentioning that we performed similar experiments with higher initial PPA coverages (0.15 and 0.39 ML after 75 °C) and the corresponding RER curves are also presented in part a of Figure 1. The final RER value after 450 °C increases linearly with the coverage, reaching a maximum value of 2.2 for 0.39 ML PPA, indirectly confirming the formation of the saturated molecular adlayer. We conclude that the increase in RER is due to the presence of adsorbed PPA molecules, i.e. the higher the coverage, the greater the reduction of the oxide surface. The contribution of the clean oxide surface reduction, i.e. without any PPA, to this value can be found in the literature and does not exceed RER 0.1 after final annealing.^40^

The corresponding resonance enhancement values D­(Ce^4+^) and D­(Ce^3+^) decreased and increased, respectively, after PPA deposition (Figure 1b, points at 25 °C). Specifically, the similar increase in D­(Ce^3+^) and decrease in D­(Ce^4+^) are expected to result from the electronic reduction of the oxide surface, since water desorption is negligible due to the minimal adsorption of OH^–^ groups on the CeO_2_ surface.^41^ In reality, the magnitude of this change is different due to attenuation by the molecular adlayer, which further reduces both signals. During thermal treatment, the behavior of both signals is quite stable up to 225 °C. Then, the D­(Ce^3+^) value starts to increase rapidly while the D­(Ce^4+^) value remains without significant changes, in accordance with the RER behavior. This indicates the onset of the surface reaction, which may include PPA decomposition/desorption and water desorption.

For CeO_1.7_ (Figure 1d), which is an intermediate case between CeO_2_ and Ce_2_O_3_, the PPA adsorption leads to surface reduction (RER change from 5.6 to 6.7). The RER remains stable after heating to 75 °C, and the surface oxidation starts at 150 °C annealing and it is very pronounced up to 300 °C (RER change from 6.0 to 4.0). The corresponding D­(Ce^4+^) curve slightly increases and D­(Ce^3+^) decreases over the whole temperature range (Figure 1e). This is due to two factors: the attenuation caused by the PPA adlayer and the possible incorporation of PPA oxygen atoms into the lattice structure of the oxide film, i.e., surface oxidation.

In the case of Ce_2_O_3_ (Figure 1f), the resonance intensity D­(Ce^3+^) decreased significantly after PPA deposition, similarly to the PPA/CeO_1.7_ system. During annealing, the D­(Ce^3+^) signal continues to decrease, is stable in the range of 150–350 °C, and then a slight increase is observed with an absolute value similar to that after PPA adsorption.

A similar behavior was observed for the Ce_6_WO_12_ oxide (Figure 1c). Here, the intensity of the D­(Ce^3+^) signal also decreased significantly after PPA deposition. The main reason for this is the attenuation of the signal by the molecular adlayer, since surface oxidation is not expected due to the absence of oxygen vacancies (note that the PPA coverage is about twice that of PPA on other oxide films, Table 1). With increasing temperature, the signal is stable up to 300 °C. Above this temperature, the D­(Ce^3+^) value increases (Figure 1c). After final annealing at 450 °C, the signal tends to return to its original value before PPA deposition.

### XPS

The Ce 3d core level spectra of CeO_2_, CeO_1.7_, Ce_2_O_3_, and Ce_6_WO_12_ thin films as prepared, after PPA deposition and the thermal treatment at 450 °C are shown in Figure 2. The Ce 3d signal of the CeO_2_ clean oxide can be fitted with 3 doublets, marked with red arrows in Figure 2a, corresponding to the Ce^4+^ cations.^42^ In contrast, the Ce 3d peak for Ce_6_WO_12_ and Ce_2_O_3_ films can be fitted with 2 additional doublets (see blue arrows in Figure 2b), corresponding to the Ce^3+^ cations.^42^ For the CeO_1.7_, the Ce 3d core level can be represented as the sum of Ce^4+^ and Ce^3+^ components, as shown in Figure S4 of Supporting Information. The PPA deposition has little effect on the Ce 3d core level shapes for all oxides. The most pronounced changes were detected for the PPA/Ce_2_O_3_ system at 25 °C as the appearance of a small Ce^4+^ related signal at 917.3 eV (Figure 2d), confirming partial oxidation of the film through its thickness. The final annealing at 450 °C visibly changes the Ce 3d core level for PPA adlayers on CeO_2_, CeO_1.7_, and Ce_2_O_3_ films while that for the PPA/Ce_6_WO_12_ remains without new components. In particular, the spectrum shows signs of film reduction (filling of the valley at 885 eV) for PPA/CeO_2_ and film oxidation (further growth of the signal at 917.3 eV) for PPA/CeO_1.7_ and PPA/Ce_2_O_3_.

**Figure 2:**
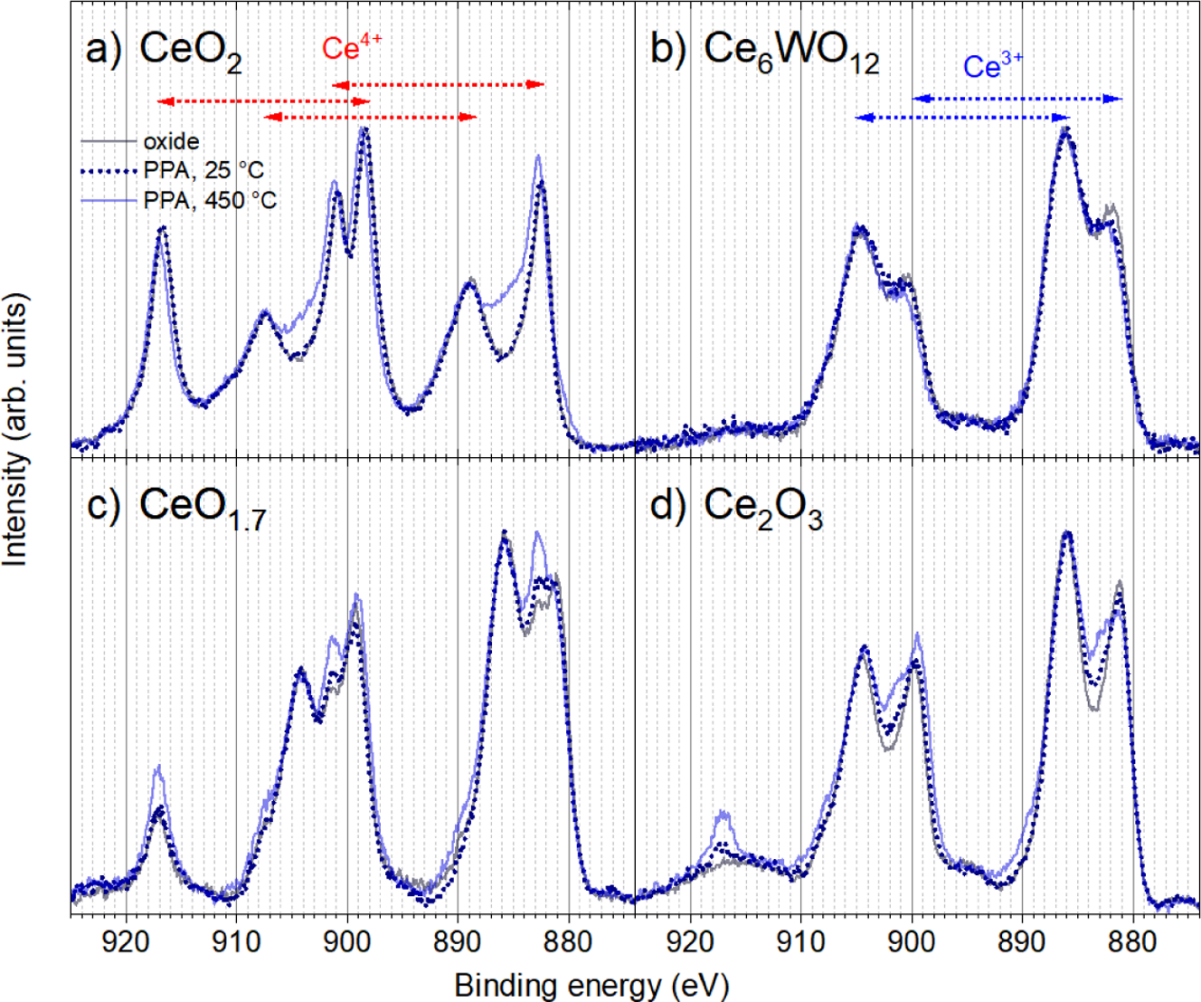
Ce 3d core level spectra of cerium oxide films (a) CeO_2_, (b) Ce_6_WO_12_, (c) CeO_1.7_, and (d) Ce_2_O_3_ before and after PPA deposition, and after annealing at 450 °C. Spectra are normalized to the maximum intensity. The photon energy is 1486.6 eV.

### SRPES

#### PPA Adsorption on CeO_2_, CeO_1.7_, Ce_2_O_3_, and Ce_6_WO_12_

The spectral changes of the P 2p, C 1s, and O 1s core levels for the PPA/CeO_2_ system after molecular deposition and during thermal treatment are shown in parts a, b, c, respectively, of Figure 3. The corresponding intensity curves are shown in Figure 4a. Note that the O 1s (PPA) signal intensity (Figure 4a) is the intensity of the PPA-induced shoulder in the O 1s region only, without the lattice component (O_latt_). After PPA deposition, the P 2p region can be represented by a 2p spin–orbit split doublet with the 2p_3/2_ component at 132.6 eV, assigned to the PO_3_^*x*–^ groups in mono- or diprotic state Our data on the BE of the P 2p core level are in good agreement with values for other systems. In particular, for a PPA adlayer on TiO_2_ ^9,13^ and ZnO^17^ oxide surfaces, the P 2p core level of the fully deprotonated PO_3_^2–^ group in bidentate geometry appears with the main doublet component in the range of 133.2–133.9 eV. A lower BE of 132.2 eV was observed for the fully deprotonated PPA molecular adlayer in tridentate geometry on the Cu(111) surface.^43^

**Figure 3:**
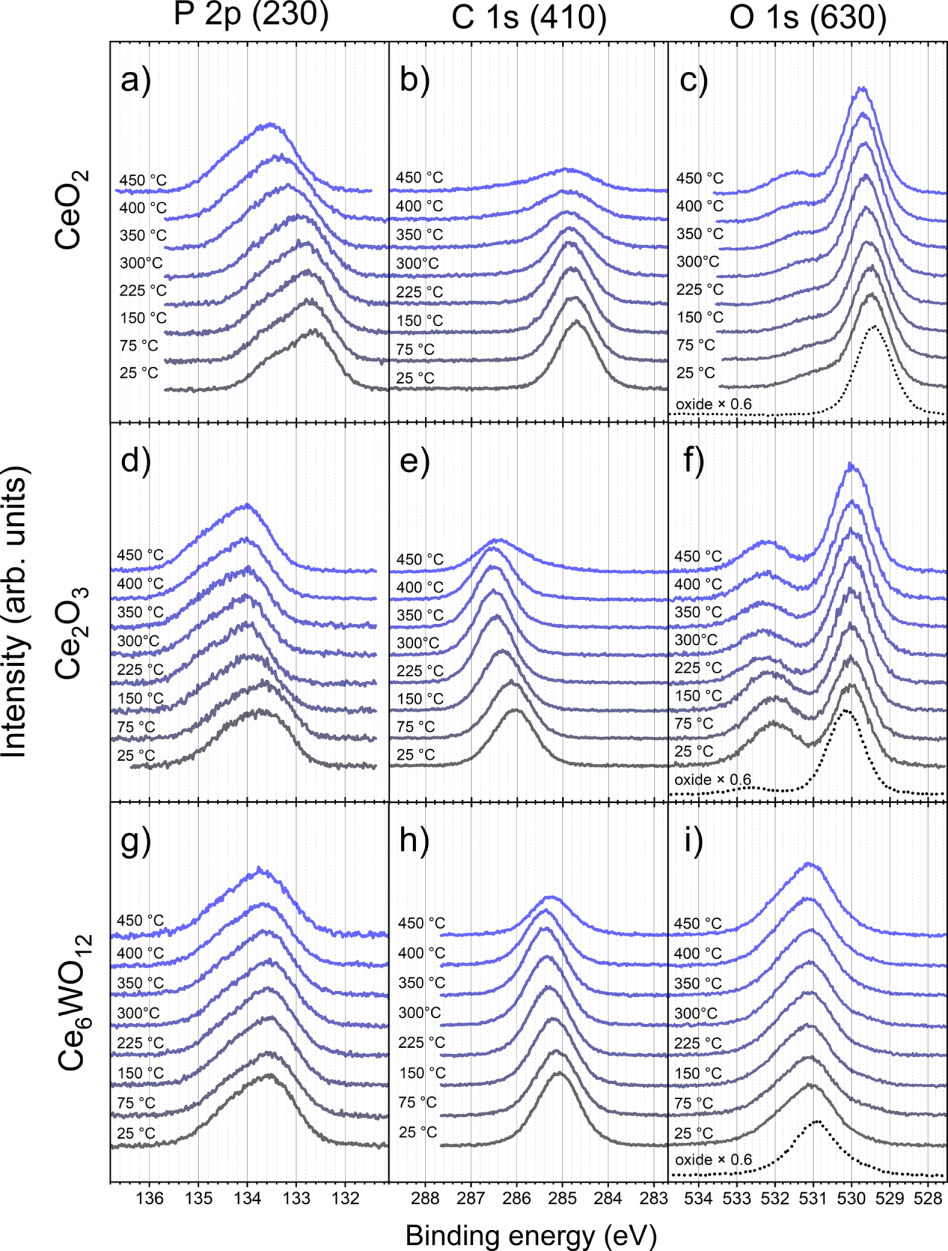
Evolution of P 2p, C 1s, and O 1s core level spectra after PPA deposition on (a–c) CeO_2_, (d–f) Ce_2_O_3_, and (g–i) Ce_6_WO_12_ as a function of temperature, measured with photon energies of 230, 410, and 630 eV, respectively. The black dotted lines correspond to the O 1s signals from the clean oxide films.

**Figure 4:**
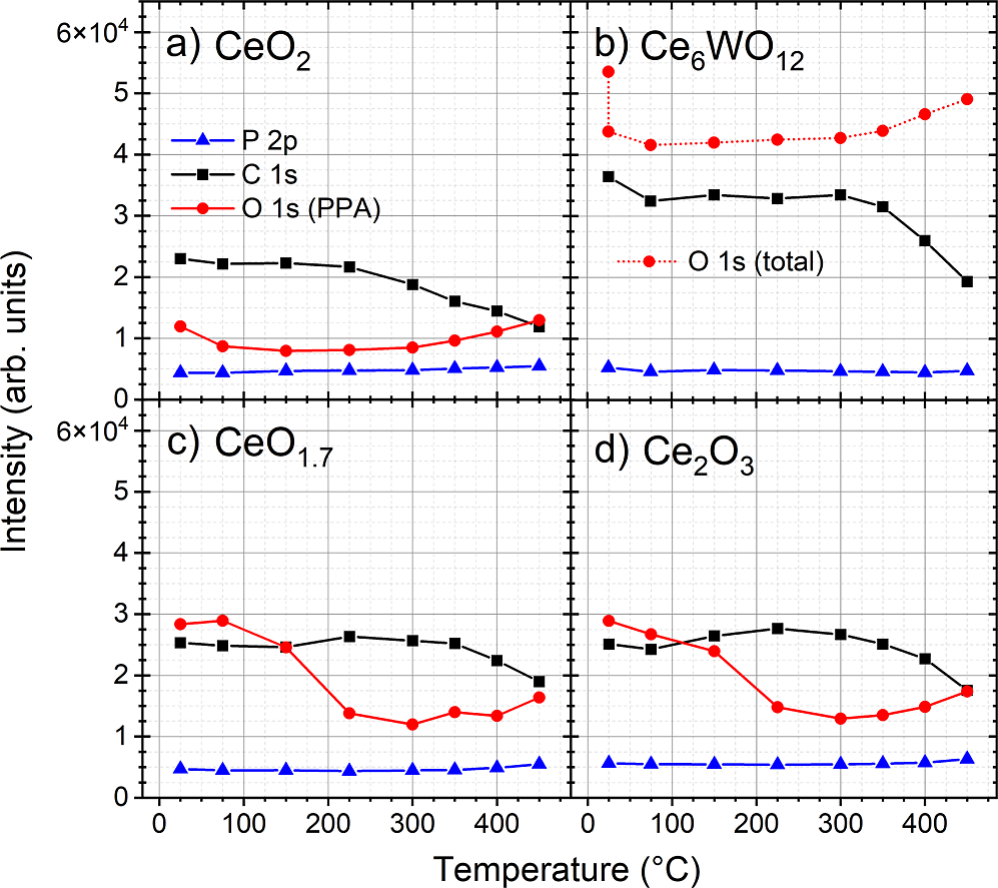
P 2p (blue), C 1s (black), and O 1s (red) core level intensities during the annealing of (a) PPA/CeO_2_, (b) PPA/Ce_6_WO_12_, (c) PPA/CeO_1.7_, and (d) PPA/Ce_2_O_3_ systems. Note that the PPA-induced O 1s signal (O 1s (PPA), red) accounts for the high binding energy component of the O 1s peak, while the total O 1s intensity (O 1s (total), dotted red) is plotted for Ce_6_WO_12_.

The adsorbed PPA adlayer is characterized by a single C 1s core level component with a BE of 284.7 eV (Figure 3b), which was assigned to the carbon atoms of the phenyl ring, in agreement with published data.^9,13^

The O 1s spectrum of the clean CeO_2_ film was dominated by a peak at 529.4 eV assigned to O^2–^ anions in the oxide lattice (see SRPES spectra in Figure 3c and XPS spectra in Figure 5a). The quality of the CeO_2_ film was confirmed by the stable O 1s signal in UHV without visible intensity due to OH^–^ groups at higher BE than the lattice peak, a sign of the presence of Ce^3+^ cations on the surface. After PPA deposition, a shoulder appeared at about 530.8 eV, which can be fitted with several components, including P–O–Ce, P–O–H, P=O, and OH^–^ groups. The variety of components is supported by the literature data on the BE separation between the lattice oxygen peak and the PPA-related adsorbed species. Specifically, the metal–O–P, P=O, and P–OH components are approximately 1, 2, and 3 eV away from the lattice oxygen peak, respectively.^9,13,17^ A contribution of adsorbed OH^–^ groups is also expected.^41^ Due to the low intensity of the PPA-induced shoulder in the O 1s core level spectra, an unambiguous assignment of this component is not possible.

**Figure 5:**
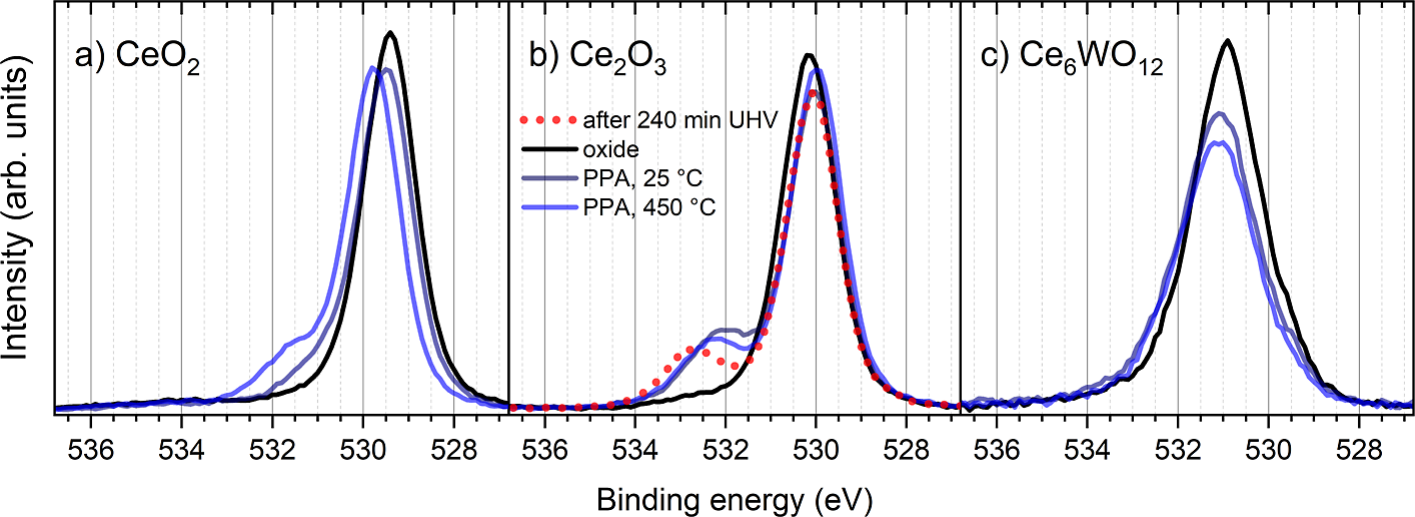
O 1s core level spectra of (a) PPA/CeO_2_, (b) PPA/Ce_2_O_3_, and (c) PPA/Ce_6_WO_12_ systems at the indicated experimental steps. The photon energy is 1486.6 eV.

Thermal treatment of PPA adlayer on CeO_2_, CeO_1.7_, Ce_2_O_3_, and Ce_6_WO_12_.

For the PPA/Ce_2_O_3_ system, the P 2p, C 1s, and O 1s molecular signals appeared at 133.5, 286.1, and 532.0 eV (Figure 3d–f), and are assigned, as before, to the adsorbed phosphonic groups, the carbon atoms of the PPA phenyl ring, and the PPA oxygen atoms, respectively. The corresponding intensity behavior is shown in Figure 4d. The shift of all spectra to high BE up to 0.9 eV compared to the PPA/CeO_2_ system is related to the semiconducting nature of the substrate and has been previously observed for similar adsorbed species on different cerium oxide films.^24,44,45^ The shape of the P 2p peak is not as well-defined as in the case of PPA/CeO_2_, indicating the presence of several types of PO_3_^*x*–^ states corresponding to different molecular binding to the surface (Figure 3d). The C 1s core level is a single peak, as expected for the PPA adlayers (Figure 3e). The O 1s spectrum before PPA deposition consists of two peaks at 530.1 and 532.6 eV, which are attributed to O^2–^ anions in the oxide and adsorbed OH^–^ groups, respectively (see SRPES spectra in Figure 3f and XPS spectra in Figure 5b).^42^ The reduced cerium oxide film itself is very reactive in UHV and actively decomposes water molecules from the residual gas.^41^ This can be seen by following the growth of the component at 532.6 eV after 240 min in UHV (Figure 5b). Before the PPA deposition, the substrate was again flashed at 550 °C and the corresponding O 1s spectrum, defined as “oxide” in Figure 5b, was a clean surface signal with a minimal amount of the adsorbed OH^–^. After PPA deposition, a new component appeared at about 532.0 eV. Note that the intensity of the component at 532.0 eV is shown as the O 1s (PPA) signal in Figure 4d. It may contain signals from the various species listed above. It is worth noting that the BE of the OH-related shoulder (532.6 eV) is slightly higher than the PPA-induced signal (532.0 eV), a clear indication of a chemically different adlayer. In addition, the intensity of the PPA-related signal is even higher, indicating the preferential adsorption of PPA molecules during deposition (in the preparation chamber with an order of magnitude higher pressure than in the analysis chamber) and the blocking of surface-active sites for OH^–^ groups, demonstrating the protective properties of PPA molecules.

The P 2p, C 1s, and O 1s spectra for the PPA/CeO_1.7_ system are presented in Figure S5 of Supporting Information as they are very similar to the PPA/Ce_2_O_3_ case. The intensity behavior of the core levels during the annealing is shown in Figure 4c, and again all curves resemble PPA on the Ce_2_O_3_ film.

Similarly, for the PPA/Ce_6_WO_12_ system, the P 2p and C 1s core levels at 133.5 eV (Figure 3g) and 285.1 eV (Figure 3h) were assigned to the PO_3_^*x*–^ groups and the carbon atoms in the phenyl ring, respectively. The O 1s peak of the clean substrate was a single peak at 530.9 eV formed by the O^2–^ anions in the oxide lattice (Figure 3i).^39^ The Ce_6_WO_12_ film was stable in UHV with no visible signal due to OH^–^ adsorption, similar to the CeO_2_ case. After PPA deposition, a shoulder at higher BE appeared. Note that the integrated intensity of the lattice and PPA oxygen signals is shown as the O 1s (total) curve in Figure 4b, because it was not possible to unambiguously separate these components. Again, as described above, several components originating from the adsorbed species can be expected in the PPA-induced shoulder in O 1s.

#### Thermal treatment of PPA adlayer on CeO_2_, CeO_1.7_, Ce_2_O_3_, and Ce_6_WO_12_

The thermal treatment of the PPA/CeO_2_ system can be divided into two temperature ranges: 25–225 and 225–450 °C. During the annealing steps from 25 to 225 °C, the spectra did not change significantly (Figure 3a–c). After 75 °C treatment, the intensity of the C 1s and O 1s peaks decreased slightly, while P 2p remained unchanged (Figure 4a). Desorption of weakly bound species and rearrangement of molecular adlayers are possible explanations for the observed behavior, which result in a change of effective thickness from 0.22 to 0.27 nm (Table 1). For the lower temperature range, the PPA-related P 2p, C 1s, and O 1s peaks were shifted to higher BE by 0.2, 0.1, and 0.2 eV, respectively. The shape of the C 1s and O 1s spectra remained the same, while the P 2p doublet became broader (Figure 3a–c). The C 1s intensity was stable up to 225 °C, while the intensity of P 2p and O 1s increased slightly (Figure 4a).

In the higher temperature range of 225–450 °C, the spectra showed more significant changes. The P 2p core level shifted by another 0.7 eV toward higher BE after heating to 450 °C, with the final position of the 2p_3/2_ peak at 133.4 eV (Figure 3a). The intensity of the P 2p doublet also increased slightly during annealing (Figure 4a). The intensity of the C 1s peak decreased in this temperature range (Figure 4a) with the appearance of a shoulder at about 286.3 eV after annealing at 350 °C (Figure 3b). The P 2p and C 1s changes are related to the decomposition of PPA with the appearance of modified phosphonic groups and new carbonaceous species on the surface. The PPA-related component in the O 1s spectra also reflects the surface chemistry in the PPA adlayer: the shoulder of increasing intensity (Figures 4a and 5a) is shifted by 0.5 eV to high BE after 450 °C treatment (Figure 3c).

The thermal treatment of the PPA/Ce_2_O_3_ system can be divided into three temperature ranges: 25–75, 75–300, and 300–450 °C. After initial annealing at 75 °C, the intensity of all core levels decreased slightly (Figure 4d), while the shapes remained unchanged (Figure 3d–f). For the 75–300 °C range, the P 2p, C 1s peaks, and the PPA-related component in O 1s were shifted by 0.4, 0.4, and 0.3 eV, respectively, to higher BE with a better resolved P 2p doublet (Figure 3d–f). The intensity of P 2p was stable, C 1s increased while that of O 1s decreased (Figure 4d). All these changes were attributed to the rearrangement of the PPA adlayer, which is consistent with the change in effective thickness from 0.17 to 0.35 nm after 75 °C (Table 1). During the further annealing from 300 to 450 °C, the peak positions remained unchanged, while the intensity of C 1s decreased and that of P 2p and O 1s increased (Figure 4d). Thus, the amount of carbonaceous species is lower at the end, probably due to PPA decomposition with C–P bond cleavage. The position and shape of the PPA-related shoulder in O 1s after 450 °C indicates the absence of adsorbed OH^–^ groups and the presence of phosphonate species (Figure 5b). The core level spectral shapes (Figure S5) and corresponding intensity curves (Figure 4c) for the PPA/CeO_1.7_ system during thermal treatment are very similar to the PPA/Ce_2_O_3_ case.

During the annealing of the PPA/Ce_6_WO_12_ system, the PPA adlayer showed the most stable behavior compared to the previous substrates. Two temperature ranges can be identified: 25–300 and 300–450 °C. After 75 °C treatment, the intensity of all core levels slightly decreased due to the PPA desorption/rearrangement, then the peak shapes and intensities remained almost unchanged up to 300 °C (Figure 4b). With further annealing steps, the C 1s intensity decreased rapidly, indicating molecular decomposition. Even the intensity of the P 2p peak (Figure 4b) decreased slightly, indicating partial desorption of the PPA molecules or phosphonate groups. Also, the P 2p peak gradually moved toward higher BE with a total shift of 0.1 eV after 450 °C. The shape of the O 1s spectra remained the same. The total intensity of the O 1s signal increased slightly up to 300 °C, with a more pronounced increase thereafter.

### NEXAFS

The C K-edge absorption spectra of PPA on CeO_2_, Ce_2_O_3_, and Ce_6_WO_12_ in NI, NE, and GI geometries obtained after annealing at 75 and 400 °C are shown in Figure 6. The spectrum for the PPA/CeO_1.7_ system is very similar to that for the Ce_2_O_3_ case and is shown in Figure S6 of the Supporting Information. In the C K-edge spectrum after treatment at 75 °C, the following C 1s transitions to unoccupied orbitals are identified: the highest energy C_I_, C_II_ are the main π^*^ resonance features, C_III_ is a mixed Rydberg state, C_IV_ and C_V_ are features related to σ^*^ resonances (Figure 6). In general, the shape of the C K-edge for all systems is very similar to the spectra obtained from 0.2 ML PPA/Cu(111)^43^ and 0.85 ML PPA/anatase TiO_2_(101)^9^ and represent excitations localized on the phenyl ring of PPA. The main difference is the slightly shifted peak position due to the different electronic structure of the oxide substrates. The peak positions of C_I_, C_II_, C_III_, C_IV_, and C_V_ measured after annealing at 75 °C are given in Table 2.

**Figure 6:**
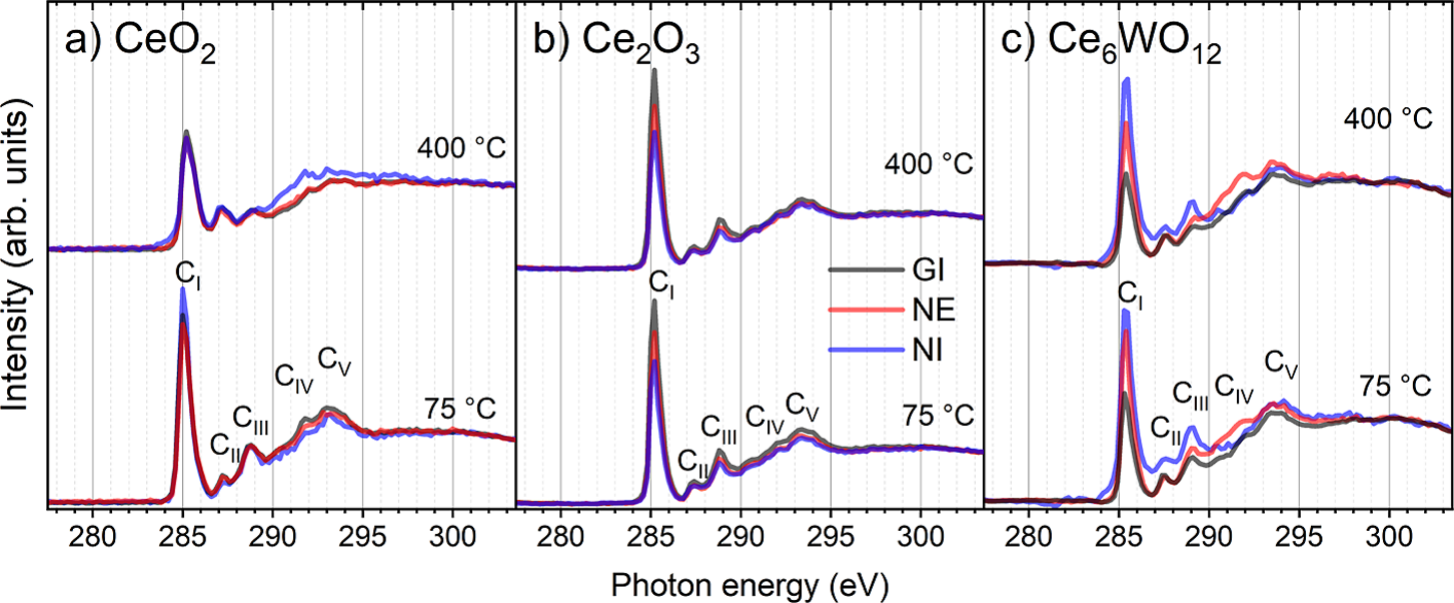
C K-edge NEXAFS spectra of PPA on (a) CeO_2_, (b) Ce_2_O_3_, and (c) Ce_6_WO_12_ measured after annealing at 75 and 400 °C. Blue, red, and black lines denote the NI, NE, and GI geometry of the substrate during the acquisition, respectively.

**Table 2:** Energies of the Peaks in the C K-Edge Spectra from PPA Adlayers on Cerium Oxide Films after Annealing at 75 °C

system	photon energy (eV)				
	C_I_	C_II_	C_III_	C_IV_	C_V_
PPA/CeO_2_(111)	285.1	287.2	288.7	291.8	293.5
PPA/CeO_1.7_	285.2	287.4	288.8	292.2	293.4
PPA/Ce_2_O_3_(111)	285.2	287.4	288.8	292.0	293.4
PPA/Ce_6_WO_12_(100)	285.4	287.6	289.0	292.0	293.8

The intensity of the π^*^ resonance obtained at different geometries was used to estimate the angle between the phenyl ring and the surface. Using eq S3 in Supporting Information, the estimated angles are 58°, 50°, 50°, and 62° for PPA on CeO_2_, CeO_1.7_, Ce_2_O_3_, and Ce_6_WO_12_, respectively, after annealing at 75 °C. The annealing of PPA/CeO_2_ at 400 °C resulted in lower intensity of the π^*^ resonance and less defined structure of the C_IV_ and C_V_ peaks. In the case of annealing of PPA on Ce_2_O_3_ and Ce_6_WO_12_ at 400 °C, the spectra remained without significant changes (Figure 6).

### DFT

The optimized adsorption configuration for a PPA molecule has the phenyl ring in an upright position, the two acidic H located above adjacent O_latt_ sites, and the phosphoryl P=O located above the Ce atom between these sites, with Δ*E*_ads_ of −1.24 eV (PBE; Figure 7a) or −1.85 eV (optB86b), respectively. This interaction being electrostatic or possibly redox in nature is evidently much stronger than PPA adsorbed on Cu(111), where Δ*E*_ads_ is −0.39 eV (PBE).^43^ The basic nature of O_latt_ means the dissociation of the acidic hydrogen is favorable. At 1/9 ML, dehydrogenation occurs consecutively without a stable monoprotic intermediate state to produce phenylphosphonate (C_6_H_5_PO_3_, abbreviated as PP) and the two acidic H atoms adsorbed onto O_latt_ sites

$$C_{6}H_{5}{PO}_{3}H_{2}\to PP+2H$$

1 $$\left(\Delta E_{rxn}=-0.48\;eV\;\left(PBE\right);-0.41\;eV\;\left(optB86b\right)\right)$$

**Figure 7:**
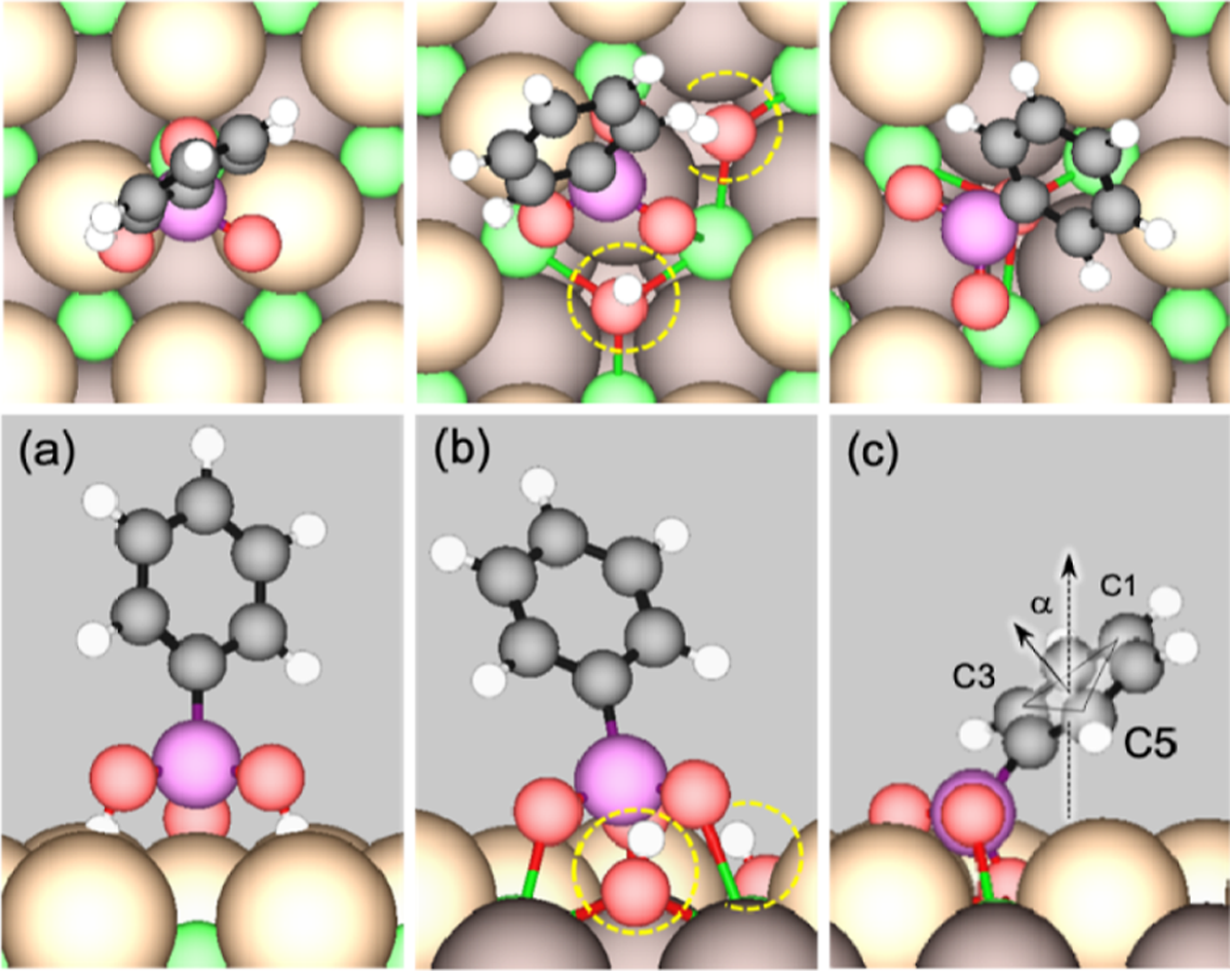
Top (top panels) and side (bottom panels) of GGA-PBE minimum-energy adsorption configurations on CeO_2_(111) of: (a) molecular PPA; (b) PP + 2H; (c) PP/V_O_. The tilt angle, α, and the C1, C3, and C5 atoms are labeled in (c). Color code: green = Ce, light brown = surface O_latt_, dark brown = subsurface O_latt_, red = O in adsorbate (including lattice-embedded OH^–^ groups, circled in (b)), violet = P, black = C, and white = H.

The PP + 2H state is therefore −1.72 eV (PBE) or −2.26 eV (optB86b) with respect to gas-phase PPA. This does not preclude the possibility that a monoprotic state (C_6_H_5_PO_3_H + H) can exist at a higher PPA surface coverage. As pointed out elsewhere,^25^ an H atom on O_latt_ forms a hydroxyl group occupying a lattice site (i.e., H being equivalent to HO/V_O_). Previous surface science studies have shown that the deposition of a protic acid such as formic acid or acetic acid on CeO_2_(111) leads to the removal of O_latt_ as water in UHV, at temperatures slightly above room temperature.^46–48^ This process is postulated to occur when the anion group displaces OH^–^ to take up its lattice site.^49^ The freed OH^–^ group readily scavenges another H atom on the surface to form water. Here the displacement of OH^–^ by PP to form water is somewhat endothermic and is driven forward by desorption of water

$$PP+2H\to {PP/V}_{O}+H_{2}O↑$$

2 $$\left(\Delta E_{rxn}=+0.41\;eV\;\left(PBE\right);+0.43\;eV\;\left(optB86b\right)\right)$$

Thus, each PPA molecule adsorbed leads to the desorption of one H_2_O molecule. The major surface states of PPA on CeO_2_(111) upon annealing, therefore, are expected to consist of a mixture of PP with dissociated H atoms, and PP/V_O_ (Figure 7b,c).

The tilt angle (α) between the phenyl ring plane, defined by the plane in which the C1, C3, and C5 atoms reside and the surface plane, is computed for comparison with the NEXAFS results. The findings are tabulated in Table 3. The molecularly adsorbed PPA (Figure 7a) is nearly fully upright, PP + 2H (Figure 7b) is more tilted, and PP/V_O_ (Figure 7c) is more tilted still. α is 60.2° in the PP+2H state and 41.8° in PP/V_O_. The phenyl ring in the PPA and PP + 2H states is sufficiently far from the surface that it may rotate about the P–C bond. We have checked and found negligible effects of ring rotation on the energy and geometry of these states. The optimized tilt angles according to optB86b-vdW are similar to the PBE results.

**Table 3:** Adsorption Energy (Δ*E*_ads_, in eV) and Phenyl Ring Tilt Angle (α, in °) for DFT-Optimized PPA States on CeO_2_(111) and Ce_2_O_3_(111)

surface	state	functional	Δ*E*_ads_	α
CeO_2_(111)	PP + 2H	PBE	–1.72	60.2
		optB86	–2.26	58.0
	PP/V_O_	PBE	–5.66^a^	41.8
		optB86	–8.04^a^	37.9
Ce_2_O_3_(111)	PP + 2H	PBE	–2.09	78.3
		optB86	–2.71	78.3
	PP/V_O_^I^ + 2H	PBE	–4.04	61.4
		optB86	–4.81	60.5
	PP/V_O_	PBE	–9.74^a^	38.4
		optB86	–10.75^a^	18.7

a Δ*E*_ads_ with respect to a neutral PP group in the gas phase and surface with a V_O_. For reference, the formation energy of a surface V_O_ is +2.12, +5.45, and +5.60 eV for CeO_2_(111) and at an O_latt_^5NN^ and O_latt_^3NN^ site on Ce_2_O_3_(111), respectively, relative to gas-phase O_2_ at the given surface unit cells using GGA-PBE with *U*_eff_ = 5 eV. See Figure 8 for site designations on Ce_2_O_3_(111).

By comparing the tilt angle calculated from the intensity of the π^*^ resonance in NEXAFS (58°) and those calculated using DFT, and by assuming that what exists on the CeO_2_ surface after annealing at 75 °C is some mixture of the dehydrogenated states, we conclude that the majority state is PP + 2H with a minority of PP/V_O_ as observed in NEXAFS.

Several PPA adsorption states have also been modeled on Ce_2_O_3_(111). The native Ce_2_O_3_(111) surface has hexagonal symmetry like CeO_2_(111), but 25% of the O_latt_ sites are inherently vacant in each trilayer resulting in a cluster of V_O_ (Figure 8a), hereafter labeled V_O_^I^ to distinguish them from oxygen vacancies that form due to the removal of existing O_latt_, e.g., via water formation. The PPA states considered include: PP+2H with PP located on oxidized sites (Figure 8b); PP + 2H with PP occupying one of the surface V_O_^I^ (Figure 8c); and PP occupying a surface V_O_ created following water formation and desorption (Figure 8d). On the Ce_2_O_3_(111) surface, there are two different O_latt_ sites (those with 5 nearest-neighbor (NN) O_latt_ in the surface layer, and those with 3) and two different V_O_^I^ sites (those with 5 NN O_latt_ in the surface layer, and those with 3 in each V_O_^I^ cluster; labeled in Figure 8a). The adsorption energy of PPA varies somewhat between the different O_latt_ sites and between the different V_O_^I^ sites. The main difference is, however, due to whether PP is located on oxidized sites or occupies a V_O_. Below we examine the energetics of these three states based on PPA adsorption around the O_latt_^5NN^ site as labeled in Figure 8a.

**Figure 8:**
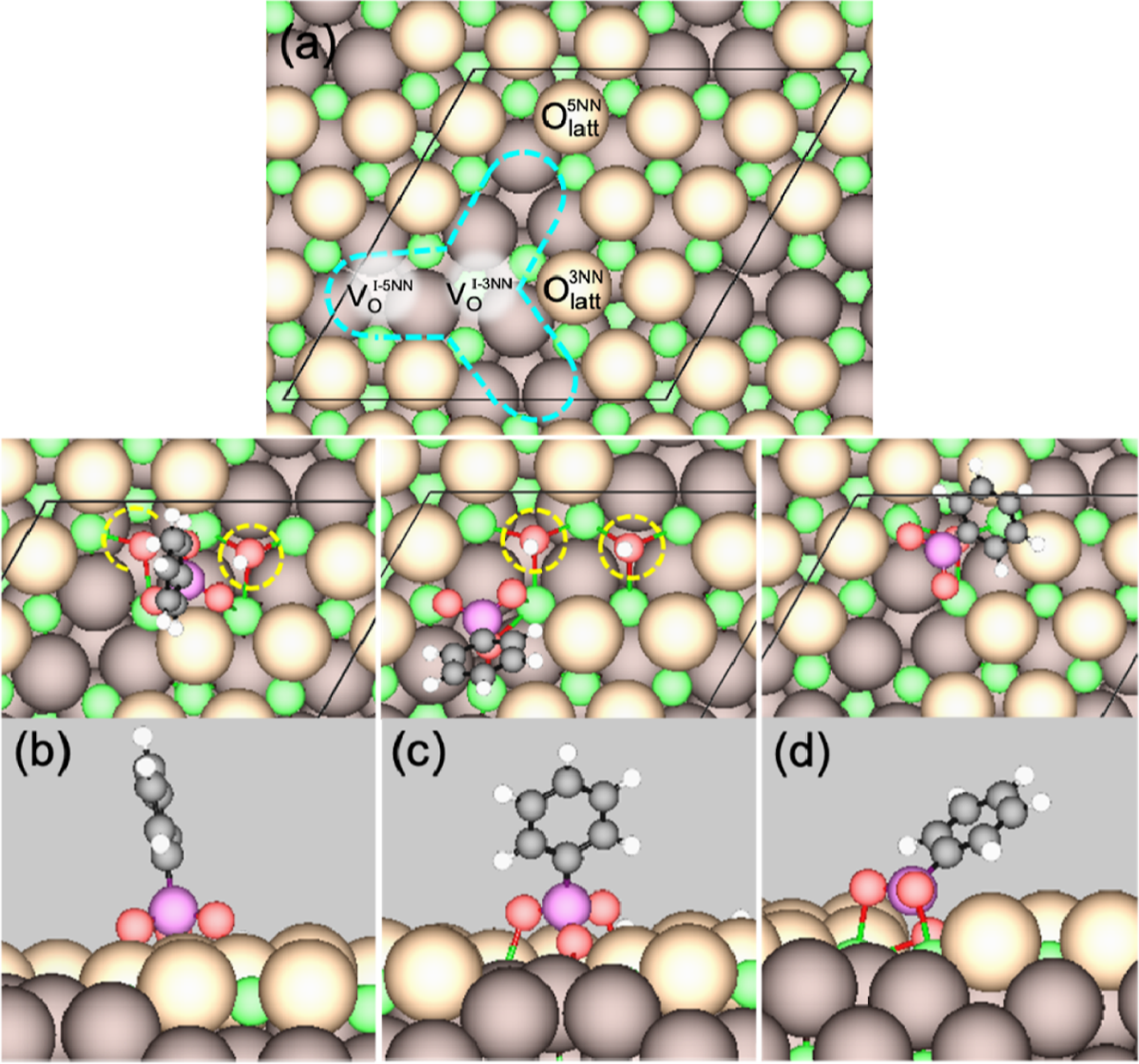
(a) Top view of the Ce_2_O_3_(111) surface with the (4 × 4) unit cell outlined by solid black lines and the surface V_O_^I^ cluster outlined by blue dashed lines. Different O_latt_ and V_O_^I^ sites are labeled. Top (top panels) and side (bottom panels) of GGA-PBE minimum-energy adsorption configurations on Ce_2_O_3_(111) of: (b) PP+2H (H on O_latt_^5NN^); (c) PP/V_O_^I‑5NN^ + 2H (H on O_latt_^5NN^); and (d) PP/V_O_ (V_O_ on O_latt_^5NN^). Color code: green = Ce, light brown = surface O_latt_, dark brown = subsurface O_latt_, red = O in adsorbate (including lattice-embedded OH^–^ groups, circled in (b,c)), violet = P, black = C, and white = H.

When PPA dissociatively adsorbs on oxidized sites of Ce_2_O_3_(111) with its acidic H atoms transferred to O_latt_^5NN^ sites (PP + 2H, Figure 8b), the Δ*E*_ads_ is −2.09 eV (PBE) or −2.71 eV (optB86b) compared to −1.72 eV (PBE) and −2.09 eV (optB86b) respectively on CeO_2_(111) (Figure 7b). Afterward, the PP group should preferentially diffuse to and occupy a surface V_O_^I^ site (PP/V_O_^I^ + 2H; Figure 8c) because the energetic driving force is enormous

$$PP+2H+V_{O}^{I}\to {PP/V}_{O}^{I}+2H$$

3 $$\left(\Delta E_{rxn}=-1.94\;eV\;\left(PBE\right);-2.10\;eV\;\left(optB86b\right)\right)$$

The reduction of an oxidized site on Ce_2_O_3_(111) via water formation and desorption is somewhat more favorable than eq 2 on CeO_2_(111)

$$PP+2H\to {PP/V}_{O}+H_{2}O↑$$

4 $$\left(\Delta E_{rxn}=+0.01\;eV\;\left(PBE\right);+0.16\;eV\;\left(optB86b\right)\right)$$

Clearly this step is not competitive against eq 3. Therefore, we do not expect eq 4 to occur until V_O_^I^ sites are saturated. Neither eqs 3 nor [Disp-formula eq4] results in the oxidation of any Ce^3+^ to Ce^4+^ based on VASP analyses of *lm*-decomposed site-projected charge and spin densities.

As on CeO_2_, PPA adsorbed on the oxidized sites has the most upright geometry. The V_O_^I^ cluster can accommodate a PP group tilted at various angles with respect to the surface (not shown) due to the more open space. Finally, the most tilted state is PP/V_O_ (Figure 8d). We surmise that the tilt angle deduced from NEXAFS for PPA on Ce_2_O_3_ (50°) reflects an approximately equal mixture of PP/V_O_^I^ + 2H and PP/V_O_ states after annealing at 75 °C. A higher proportion of PP/V_O_ is consistent with water formation due to O_latt_ displacement by a PP group (eq 4) being more facile on Ce_2_O_3_(111) than on CeO_2_(111) (cf. eq 2).

## Discussion

The cerium oxides used in this study differ in the concentration of surface vacancies, stoichiometry and composition. The adsorption of PPA molecules on the CeO_2_ film (film with Ce^4+^ cations in the absence of oxygen vacancies) resulted in a minor reduction of the surface, as evidenced by an RER value of 0.05 (Figure 1a), without modifying the bulk electronic structure of the oxide (Figure 2a). We suggest that the reduction is electronic in nature at this temperature, similar to that observed for adenine on CeO_2_,^23^ since water desorption is minimal.^41^ Furthermore, using the procedure described in ref 50, based on the proportionality of the RER value to the ratio of Ce^3+^ and Ce^4+^ centers on the surface with a coefficient of 5.5, the charge transfer from PPA to the surface is estimated to be 0.1 e^–^ per molecule (for details see Section S8 of Supporting Information). The complete deprotonation of the phosphonic group after the adsorption, i.e. forming adsorbed PP species, is expected and confirmed by the DFT calculations (Figure 7b). There has been no model study investigating the adsorption of phosphonic acids on CeO_2_ surfaces, but for phosphates (−OPO­(OH_2_)) the deprotonation on CeO_2_(111) has been predicted by DFT^25^ with the most stable tridentate bonding configuration.^26^ As a result, the acidic hydrogen atoms likely accumulate on the surface, forming surface-embedded OH^–^ groups. Water desorption cannot be completely excluded, but is contradicted by the small surface reduction. We conclude that PPA molecular adsorption is accompanied by the accumulation of hydroxyl groups on the surface of CeO_2_ (see schematic representation in Figure 9).

**Figure 9:**
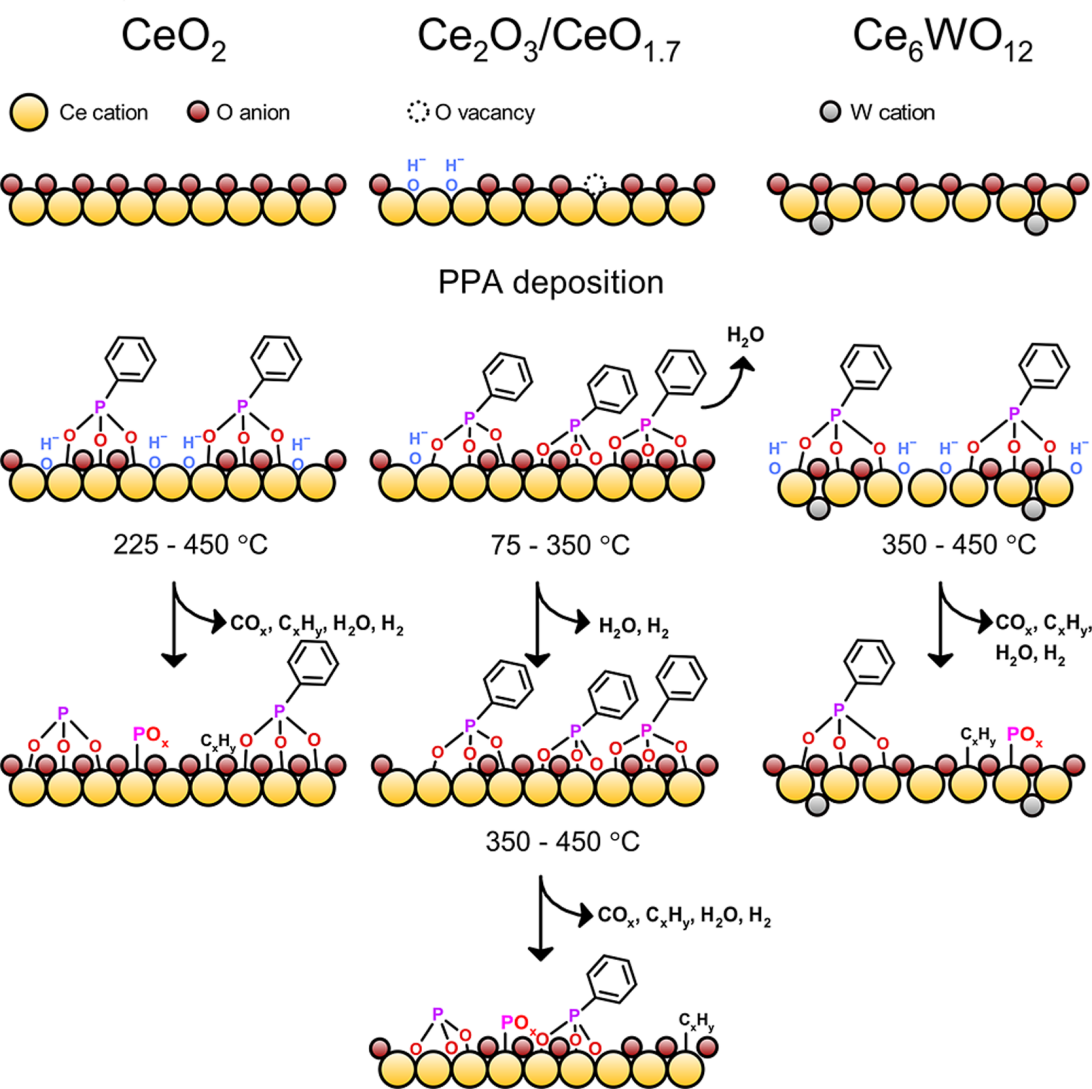
Schematic illustration of the thermal decomposition of PPA on CeO_2_, Ce_2_O_3_, CeO_1.7_, and Ce_6_WO_12_ upon annealing. The proposed desorption products are shown for each temperature range.

Surface reduction is also observed for the PPA/CeO_1.7_ system with both Ce^4+^ and Ce^3+^ cations, after the PPA deposition, giving an RER change of 1.1 (Figure 1d), which is much higher than for the CeO_2_ film. Applying the previously introduced proportionality between RER and the concentration of different cerium cations on the surface,^50^ we estimated that the ratio of the adsorbed PPA to the desorbed H_2_O molecules is expected to be about 1:1 (for details see Section S8 of Supporting Information), suggesting that each H_2_O molecule is formed from the two acidic H atoms of a PPA molecule. This leads to the idea of preferential adsorption of PPA molecules, which expel adsorbed OH^–^ groups from the surface in the form of water as calculated theoretically by eq 2 above. Since the reduced cerium oxide surface is enriched with OH^–^ groups in UHV, the protons from the adsorbed PP combine with surface OH^–^ and desorb as water, creating vacancies V_O_. The electronic reduction mechanism cannot be excluded, but we expect it to make only a small contribution, based on the behavior of the PPA/CeO_2_ system at 25 °C. The bulk electronic structure remains unaffected after the PPA adsorption on CeO_1.7_ (Figure 2c). Again, complete deprotonation of the phosphonic group occurs upon adsorption, with the P–O groups being trapped by the available vacancies.^25^ Molecular adsorption with P–O bonds on top of cerium cations and filling the vacancies V_O_^I^ and V_O_, explains the lower tilt angle (50°) of the phenyl ring with respect to the surface plane for reduced oxides compared to 58° and 62° for PPA on CeO_2_ and Ce_6_WO_12_ oxides, respectively.

The D­(Ce^3+^) curves for CeO_1.7_ and Ce_2_O_3_ oxides show a decreasing trend over almost the entire temperature range, indicating surface oxidation (Figure 1e,f). However, the RERs for the PPA/CeO_1.7_ case clearly show a reduction after the PPA adsorption, so the reduction cannot be excluded for PPA/Ce_2_O_3_ either. It is likely that surface reduction (in terms of oxygen loss from the surface) competes with surface oxidation (vacancy filling by PPA or interaction with water from the residual atmosphere) on the surface with the high vacancy concentration. From the analysis of the Ce 3d core level spectra, it can be concluded that the dominant process is oxidation of the Ce_2_O_3_ film after PPA adsorption, which increases the Ce^4+^ concentration in the subsurface region of the oxide (Figure 2d). Combining the results for the CeO_1.7_ and Ce_2_O_3_ substrates, the schematic representation of PPA adsorption on Ce_2_O_3_ (composed of Ce^3+^ cations and a maximum number of oxygen vacancies) is shown in Figure 9.

The electronic structure of the Ce_6_WO_12_ oxide containing only Ce^3+^, as judged by the Ce 3d (Figure 2b) and O 1s (Figure 5c) signals, does not change much after PPA deposition. The D­(Ce^3+^) value (Figure 1c) drops significantly after PPA adsorption but in this case, we assume that it is mostly attenuated by the molecular adlayer, although the expected initial oxide reduction cannot be excluded. Again, tridentate molecular adsorption appeared, accompanied by the formation of OH^–^ groups (see schematic representation in Figure 9).

The analysis of the P 2p, C 1s, and PPA-related feature, i.e. the high BE shoulder, in the O 1s core level (Figure 3) together with the P:C stoichiometry ratios (Table 1) allows us to conclude that the adlayer consists of the PPA molecules adsorbed on the surface via the phosphonate group. The adsorption geometry is most probably tridentate at low molecular coverage, but it is difficult to determine accurately from the SRPES data due to the ambiguity of the P 2p peak (Figure 3a,d,g). Similarly, the O 1s shoulder cannot be fitted into individual components related to the phosphonate oxygen atoms, which may be partially or fully deprotonated (Figure 3c,f,i). The single component C 1s, characteristic of all three systems, appeared at slightly different BE due to the semiconducting nature of the oxide film (Figure 3b,e,h). The similar shape of the C 1s and the intensity (Figure 4), which is proportional to the effective PPA thickness or coverage (Table 1), reflect the fact that the phenyl ring is not involved in binding to the oxide surface. Since the as-deposited molecular films may contain the physisorbed species, quantification was also performed for the adlayers after the first annealing step at 75 °C (Table 1). The thermal treatment provides additional energy to the system, which stimulates molecular diffusion on the surface, water desorption, etc., affecting the adsorption geometry of the molecules, while the intensity of the PPA signals changed only slightly. This is observed as an effective thickness increase for PPA on CeO_2_ and Ce_2_O_3_ substrates (Table 1).

The change in surface electronic structure during thermal treatment of the PPA/cerium oxide systems is shown in Figure 3. In particular, the P 2p core level retains its shape (Figure 3a,d,g) and intensity (Figure 4) after all experimental steps. The C 1s peak (Figure 3b,e,h) does not change during the initial stages of annealing, but then its intensity begins to decrease, and it broadens. The temperature at which this occurs is 225 °C for PPA/CeO_2_ and 350 °C for the other three systems (Figure 4). This suggests a thermally induced decomposition of the adsorbed molecules, most likely cleavage of the C–P bond and subsequent desorption of the phenyl ring-containing species and/or its fragments. It is worth noting that the C 1s intensity was reduced by 38, 11, 10 and 29% after the treatment at 400 °C for the PPA on CeO_2_, CeO_1.7_, Ce_2_O_3_, and Ce_6_WO_12_ films, respectively. In other words, CeO_2_ and Ce_6_WO_12_ films contain a lower amount of carbon species, while the oxide films with vacancies (CeO_1.7_ and Ce_2_O_3_) prevent intense molecular decomposition/desorption at this temperature.

The PPA-related O 1s component changes similarly to the C 1s signal after the initial stages of annealing (Figure 4). Then, when the decomposition temperature is reached, the O 1s intensity increases while the C 1s signal decreases. This statement is fully valid for the PPA/CeO_2_ and PPA/Ce_6_WO_12_ systems. In the case of PPA on CeO_1.7_ and Ce_2_O_3_, the intensity of the PPA-related O 1s component decreases significantly from 75 to 300 °C, which is related to surface reoxidation, as this component also contains signal from the adsorbed OH^–^ groups.^41^ In summary, the increase of the PPA-related O 1s signal after 225 °C for PPA/CeO_2_ and after 350 °C for PPA/Ce_2_O_3_ and PPA/Ce_6_WO_12_ systems confirms the C–P bond scission and the desorption of carbonaceous species uncovering the phosphonate groups previously attenuated by phenyl rings. Within the intensity scale in Figure 4, the increase of the P 2p signal is not significant. In addition, the shape of the O 1s core level after the final treatment confirms the presence of the phosphorus species on the oxide surface (Figure 5).

The change in the oxidation state of the Ce cations on the surface during the annealing of the PPA/CeO_2_ system appears as an increase in the concentration of the Ce^3+^ centers (Figure 1a,b). Two temperature ranges can be identified. From 75 to 225 °C, the RER value obtained after the PPA adsorption is largely stable. Thus, the partial electron transfer of 0.1 e^–^ from the PPA molecules to the Ce^4+^ cations occurs mostly at the moment of molecular adsorption. The rapid increase in RER observed after 225 °C is associated with oxygen loss from the surface, which also affects the subsurface region of the oxide film (Figure 2a). It is consistent with the change in photoemission intensity discussed above, i.e., the desorption of oxygenated carbonaceous species. Desorption of water molecules cannot be excluded. It is important to note that the RER trend during the annealing is proportional to molecular coverage (Figure 1a), i.e. higher coverage results in more intense surface reduction. Again, using the linear relationship between RER and Ce^3+^/Ce^4+^ on the surface,^50^ we estimate that after the annealing the 0.07 ML PPA/CeO_2_ system at 450 °C, there is approximately 1 desorbed H_2_O per 1 PPA (for details see section S8 of Supporting Information). Following this idea, tridentate molecular coordination means the formation of two OH^–^ groups on the surface, which could desorb as a single H_2_O molecule after the final treatment. In reality, the strong reduction after 450 °C is not only due to water desorption, because we expect the desorption of oxygenated carbonaceous species too. For the 0.39 ML PPA/CeO_2_ system after 450 °C, using the same estimation, there is about 0.5 desorbed H_2_O per 1 PPA (Section S8 of Supporting Information). This analysis shows that the saturation coverage affects the bond geometry, reducing the number of P–O–Ce bonds and protecting the surface from oxygen loss. The different bonding at saturation coverage was also reflected in the changes of O 1s and P 2p core level spectra (see Figure S7a,c). The O 1s spectrum obtained at saturation coverage (Figure S7c red) contains an additional component at 532.5 eV, indicating the high contribution of P–OH bonds,^9,13,17^ consistent with phosphonate group partial deprotonation. The broader and less defined P 2p spectrum of the PPA adlayer at saturation coverage (Figure S7a red), compared to 0.07 (black) and 0.15 ML (light violet) adlayers, suggests a wider variety in phosphonate group binding geometries. No significant changes were observed in the C K-edge spectra between 0.07 and 0.39 ML PPA adlayers (see Figure S8). The only small feature that emerged was at about 290 eV for saturation coverage. The estimated tilt angle of the phenyl ring was found to be independent of PPA adlayer coverage and binding geometry, consistent with previous studies of PPA adlayers on TiO_2_.^9,13^

For the PPA/CeO_1.7_ system, the decrease of the RER value from 75 to 300 °C, i.e. the surface oxidation, goes in parallel with the decrease of the O 1s (PPA) component, which includes the signals from the PPA oxygen atoms as well as the adsorbed OH^–^ groups. Surface oxidation refers to the filling of surface vacancies with oxygen atoms from the adsorbed species, the majority of which are OH^–^ groups and PPA molecules. The decrease of the RER value by 3.8 in this temperature range allows us to estimate the oxygen gain for the surface, namely up to 30 atoms per 100 Ce^4+^ cations. Since the P 2p signal does not change significantly, i.e. there is no visible reduction of the phosphonic group, and the vacancies are expected to be filled by PPA immediately after deposition, we suggest that the surface chemistry in this region is related to the chemistry of the OH^–^ groups on the surface. Thus, surface reoxidation is believed to occur at the expense of adsorbed OH^–^ groups, in agreement with literature data on water interaction with reduced cerium oxide films.^41^

We expect the surface chemistry of the PPA adlayer on Ce_2_O_3_ to be similar to that of CeO_1.7_ oxide, with surface oxidation dominating during thermal treatment. The D­(Ce^3+^) value decreases up to 150 °C and then remains stable up to 400 °C (Figure 1f), which is explained by the filling of the oxygen vacancies on the surface by phosphonate oxygen atoms and OH^–^ groups. The final slight increase of D­(Ce^3+^) after 450 °C is due to the partial desorption of the oxygenated species from the surface. For this system, the reoxidation of the film below the surface is confirmed by the shape of the Ce 3d core level (Figure 2b).

For the PPA adlayer on Ce_6_WO_12_, the D­(Ce^3+^) value is stable during annealing and starts to increase from 350 °C (Figure 1c). For this system, D­(Ce^3+^) approaches the value of the clean surface after the final annealing, in agreement with the behavior of the O 1s (total) intensity (Figure 4b). This behavior is consistent with the formation of a stable PPA adlayer with partial C–P bond scission after 350 °C and subsequent desorption of the reaction products, leaving the electronic structure of the bulk oxide unaffected (Figure 2b).

The change in adsorption geometry was evaluated by analyzing the C K-edge NEXAFS spectra after annealing at 400 °C (Figure 6). The spectra for all systems retain their shape. The average tilt angle of the phenyl ring to the surface plane did not change significantly during the thermal treatment in the range of 75–400 °C. The intensity of the π^*^ resonances decreased only for PPA/CeO_2_ and remained unchanged for the other systems, in agreement with the C 1s data mentioned above. This indicates that PPA is thermally less stable on CeO_2_ than on the reduced surfaces. One plausible explanation is that the reduced surfaces have fewer surface oxygen sites that are involved in catalyzing C–H and P–C bond scission steps that initiate^43^ the decomposition of the phenyl group and loss of carbon from surface. These steps require a surface site to accept the dissociated H atom or C center, which is more stable when located on a lattice O than Ce.^51,52^

A schematic representation of PPA adsorption and decomposition on CeO_2_, Ce_2_O_3_, and Ce_6_WO_12_ oxides is shown in Figure 9. The molecules mostly adsorb upright with the tilted phenyl ring in a tridentate configuration at low coverage, e.g. 0.1 ML, with deprotonation of the phosphonic group and formation of surface OH^–^ groups. Water desorption is expected for the PPA/Ce_2_O_3_ system during molecular adsorption. The onset temperatures of C–P bond scission are 225 and 350 °C for the PPA adlayers on oxide films with Ce^4+^ and Ce^3+^ cations, respectively, independent of the presence of oxygen vacancies. Annealing of PPA/Ce_2_O_3_ from 75 to 300 °C has little effect on the electronic structure of the molecular adlayer and mostly oxidizes the oxide film at the expense of surface OH^–^ groups. The expected desorbing species are shown for each system.

The phosphonate anchor groups remain on the surface of all cerium oxide films after 450 °C without the appearance of new organic phosphorus species. The C–P bond scission is a common decomposition mechanism of PPA with small variations in the onset temperature.^17,43^ It is worth noting that the phosphorus species remaining on the surface after the initiation of C–P bond scission for the PPA/CeO_2_ system are characterized by the P 2p core level with a BE shift to higher values (Figure 3a): after 450 °C annealing, this shift is 0.7 eV. The shift of the O 1s (PPA) component to high BE (Figure 3c) also confirms the electronic configuration change of the phosphorus species. Similar changes of the P 2p spectra were observed earlier in the case of 0.2 ML PPA/Cu(111)^43^ after annealing at 300 °C and attributed to the PPA decomposition and formation of the (PO_3_)_*y*_ structures. Thus, (PO_3_)_*y*_ can be expected on the CeO_2_ oxide film after the annealing at 450 °C. Further DFT studies are needed to refine the mechanism of surface chemistry of PPA adlayers on ceria films, including the activation energies controlling the decomposition of PPA.

PPA mono-, bi- and/or tridentate bonding has been demonstrated for a variety of oxides, with slight variations in adsorption geometry observed as a function of molecular coverage, surface composition or thermal treatment. These systems include PPA/TiO_2_,^9,13^ PPA/Al_2_O_3_,^53^ PPA/ZnO,^17^ PPA/CoO,^12^ and PPA/Co_3_O_4_.^11,54^ Regarding the thermal stability of the PPA adlayer, molecular decomposition via C–P scission was observed for PPA/ZnO systems, starting from 280 °C.^17^ On the other hand, for PPA/TiO_2_ systems, the decomposition temperature was not reached during the thermal treatment and the molecular adlayer was shown to be stable up to 500 °C.^13^ For phosphonic acids with different functional groups on Al_2_O_3_ films, it was shown that the anchor group is stable and remains adsorbed up to 500 °C, while the functional groups decomposed below this temperature.^16^ Limited information is available for systems in which cerium oxide films are used as the substrate. Thermal decomposition of dimethyl methylphosphonate on CeO_2_(111) and CeO_*x*_ (1.5 < *x* < 2.0) oxide films proceeds first through P–O dissociation and then, above 430 °C, P–C bond scission is activated.^24^ Furthermore, the passivation of the oxide surface with PO_*x*_ species was observed after multiple cycles of dimethyl methylphosphonate adsorption and thermally activated reactions with a maximum temperature 630 °C. Molecular binding to cerium oxide was almost independent of the type of cerium cations present on the surface, similar to the present work.^24^

## Conclusions

The model systems of phenylphosphonic acid binding to different cerium oxide surfaces have been studied by photoemission-based techniques using synchrotron radiation and DFT calculations. The experimental findings can be summarized as follows:

(1) The PPA binds to CeO_2_(111), CeO_1.7_, Ce_2_O_3_(111), Ce_6_WO_12_(100) oxide films via the phosphonate group independent of surface stoichiometry and composition.
(2) The PPA adsorption geometry is mainly defined by the molecular coverage. It is tridentate for the submonolayer PPA adlayers. As coverage increases, the percentage of bi- and monodentate bound molecules increases.
(3) Bonding of PPA to the cerium oxide surface is accompanied by deprotonation of the phosphonic group and formation of the surface hydroxyl groups.
(4) The oxygen atoms of the phosphonate group as well as of the surface hydroxyl groups fill the vacancies in the oxide and promote the reoxidation of the surface and subsurface layers of the CeO_1.7_ and Ce_2_O_3_ films.
(5) The stability of the PPA adlayer is lowest for the CeO_2_ film, where the C–P bond scission starts at 225 °C. For the oxides CeO_1.7_, Ce_2_O_3_, and Ce_6_WO_12_, where the oxygen vacancies and/or Ce^3+^ cations are present, the temperature of the C–P bond scission increases to 350 °C. Thus, these temperature values define the PPA adlayer stability ranges for different cerium oxides, while the phosphorus species remain on all oxide films even after the annealing at 450 °C.
(6) DFT calculations suggest that the deprotonation of PPA is exothermic on CeO_2_(111), and it is mildly endothermic for the dissociated hydrogen atoms to remove oxygen from the oxide as water when annealed, displaced by the remaining phenylphosphonate group as it takes up the vacated lattice site. Concomitant with this sequence of steps, the phenyl ring becomes increasingly tilted toward the surface plane. On Ce_2_O_3_(111), PPA likewise prefers dissociative adsorption, with the PP group preferentially accommodated in a native oxygen vacancy with strong exothermicity. Upon saturation of the native vacancies, additional PPA would adsorb onto the oxidized site and reduce it upon annealing, yielding PP in an oxygen vacancy (PP/V_O_) and water, similarly to CeO_2_(111), albeit with somewhat more favorable energetics. Thus, a higher portion of the molecules is expected to exist as PP/V_O_ on Ce_2_O_3_(111) than on CeO_2_(111) upon annealing, which is consistent with the NEXAFS measurements of a greater tilt angle on Ce_2_O_3_(111) than on CeO_2_(111) based on the π^*^ resonance intensity.

Overall, the research provides a fundamental characterization of the adsorption and thermal stability of phenylphosphonic acid molecules on cerium oxide films. The findings will help the understanding of the phosphonic acids’ interactions with the oxide surfaces. The results will also contribute to the development of strategies for the efficient functionalization of semiconductor surfaces by phosphonic acids, advancing applications in organic electronics and creating novel materials for biomedical use.

## Supplementary Material



## Notes

A figure indicating the resonance enhancements of cerium cations in the valence band is shown. The details of the effective thickness, molecular coverage estimation, and NEXAFS data treatment are presented. The LEED patterns of different oxide films are shown. The Ce 3d, P 2p, C 1s, O 1s core level spectra, and C K-edge X-ray absorption spectra of the PPA/CeO_1.7_ system are shown. The estimation of the charge transfer from PPA and the oxygen loss from the surface is presented. P 2p, C 1s, and O 1s core levels, and C K-edge NEXAFS spectra of different PPA adlayers on the CeO_2_ oxide films are shown (PDF)
