# Supplementary material for: Adsorption and Thermal Stability of Phenylphosphonic Acid on Cerium Oxides
Source: J Phys Chem C Nanomater Interfaces. 2025 Aug 13;129(34):15265–81. doi: 10.1021/acs.jpcc.5c04065 (PMC12400423; doi:10.1021/acs.jpcc.5c04065)
Supplement: Supplementary file 1 [file jp5c04065_si_001.pdf]

Supporting Information

Adsorption and Thermal Stability of Phenylphosphonic Acid on Cerium Oxides

Viacheslav Kalinovych[1], Lesia Piliai[1], Yuliia Kosto[1], Sascha L. Mehl[2], Tomáš Skála[1], Kevin C. Prince[1,2], Iva Matolínová[1], Ye Xu[3], Nataliya Tsud[1*]

[1] Charles University, Faculty of Mathematics and Physics, Department of Surface and Plasma Science, V Holešovičkách 2, Prague, 18000, Czech Republic

[2] Elettra-Sincrotrone Trieste S.C.p.A., in Area Science Park, Strada Statale 14, km 163.5, Basovizza (Trieste), 34149, Italy

[3] Louisiana State University, Cain Department of Chemical Engineering, Baton Rouge, LA 70808, USA

[*] Corresponding author; e-mail: nataliya.tsud@matfyz.cuni.cz

S1. The resonance enhancement of cerium cations

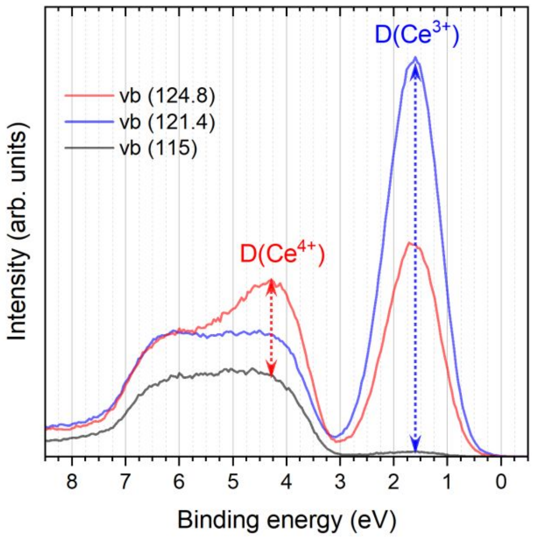

Fig. S1. Example of the valence band spectra of $CeO_{1.7}$/Cu(111) measured with a photon energy of 115, 121.4, and 124.8 eV. The resonance enhancements of Ce^{3+} and Ce^{4+} states are shown by arrows. The corresponding intensities were used to calculate the RER value.

S2. The effective thickness and coverage estimation

The effective thickness *d* of the adlayer was calculated from the equation:

$$d = \lambda_m \cdot \cos\gamma \cdot \ln\left(I_0/I_d\right), \qquad \textbf{Eq. (S1)}$$

where I_d and I_0 are the attenuated and clean surface intensity of the photoelectron signal, respectively, λ_m is the inelastic mean free path (IMFP) of photoelectrons in the adlayer material, γ is the photoelectron emission angle with respect to normal and equals 20° for XPS. The molecular coverage Θ (in MLs) was determined as the number of PPA molecules per Ce surface atom using the equation:

$$\frac{I(P\ 2p)}{I_{surf}(subs)} = \Theta \cdot \exp\left(-\frac{d_{C6H6}}{\lambda_{C_6H_6}(E_{kin}\ P2p)}\right) /$$

Eq. (S2)

$$/(1 - \Theta + \Theta \cdot \exp\left(-\frac{d_{PPA}}{\lambda_{PPA}(E_{kin}\ Ce\ 3d)}\right)).$$

The coverage calculation using **Eq. S2** was based on the following assumptions: the molecules are homogeneously distributed on the surface without multilayer formation, the PPA molecules adsorbed in an upright geometry, and the coverage Θ is proportional to the molecular signal I $(P\ 2p)$, while the "uncovered" part of the sample (1 – Θ) is proportional to the intensity of the surface substrate atoms $I_{surf}(subs)$ (**Fig. S2**). The term $\exp\left(-\frac{d_{C6H6}}{\lambda_{C6H6}(E_{kin}\ P\ 2p)}\right)$ in **Eq. (S2)** assumes the attenuation of the P 2p core level signal by the upright phenyl ring (the benzene ring was used for the calculations). Specifically, d_{C6H6} is the diameter of the benzene ring and $\lambda_{C6H6}(E_{kin}\ P\ 2p)$ is the IMFP of the P 2p photoelectrons passing through the benzene ring. The term in the denominator of **Eq. (S2)** $\Theta \cdot \exp\left(-\frac{d_{PPA}}{\lambda_{PPA}(E_{kin} Ce\ 3d)}\right)$ represents the attenuation of the substrate photoelectrons passing through the PPA molecule, where d_{PPA} is the estimated height of the PPA molecule and $\lambda_{PPA}(E_{kin}\ Ce\ 3d)$ is the IMFP of the substrate photoelectrons passing through the PPA molecule.

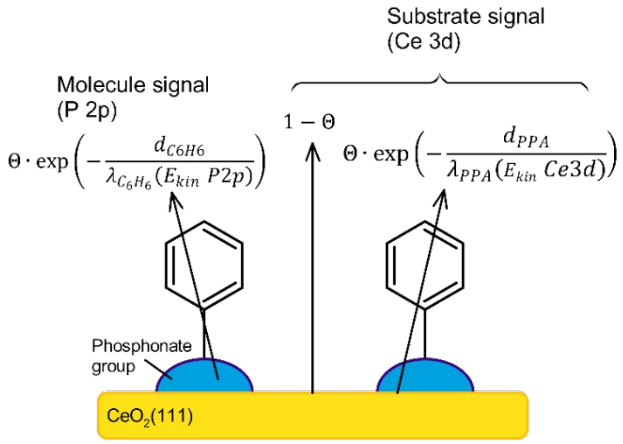

Fig. S2. Coverage estimation diagram of PPA deposited on the $CeO_2(111)$.

To calculate the intensity of the top atoms of the substrate $I_{surf}(subs)$, the following equation was used:

$$I_{surf}(subs) = I_{tot}(subs) \cdot \left(1 - \exp\left(-\frac{a_{subs}}{\lambda_{CeO2}(E_{kin}\ Ce\ 3d)}\right)\right) /$$

$$/(1 - \exp\left(-\frac{d_{subs}}{\lambda_{CeO2}(E_{kin}\ Ce\ 3d)}\right))$$

where $I_{tot}(subs)$ is the measured intensity of the Ce 3d core level substrate peak, a_{CeO2} is the thickness of the 1st substrate layer, $\lambda_{CeO2}(E_{kin}\ Ce\ 3d)$ is the IMFP of the substrate core level

photoelectrons passing through the bulk of the substrate, and d_{CeO2} is the thickness of the substrate calculated using **Eq. (S1)**. All the values for the coverage calculation are presented in **Table S1**. The molecular coverage on the $Ce_2O_3(111)$, $CeO_{1.7}$, and $Ce_6WO_{12}(100)$ were assumed to be proportional to the $I(P\ 2p)$ of PPA/$CeO_2(111)$ obtained by XPS.

Substrate parameters, nm		IMFP, nm		Molecule parameters, nm	
a_{CeO2}	0.30	$\lambda_{PPA}(E_{kin}\ Ce\ 3d)$	1.75	d_{PPA}	0.82
		$\lambda_{CeO2}(E_{kin}\ Ce\ 3d)$	1.14	d_{C6H6}	0.56
		$\lambda_{C_6H_6}(E_{kin}\ P\ 2p)$	3.54		

Table S1: Coverage calculation parameters.

S3. NEXAFS data treatment

The photoemission features in Auger-yield NEXAFS were removed by the procedure described in Ref.[1] The angular dependence of the π^* resonance intensity for the C K-edge acquired at different geometries was estimated using the following equation:[2]

$$I\ (\alpha,\beta) = K\{0.33\ P\left[1 + 0.5\ (3cos^2\beta - 1)(3cos^2\alpha - 1)\right] + 0.5\ (1 - P)sin^2\alpha\}, \qquad \textbf{Eq. (S3)}$$

where K is a constant, P is the polarization of the synchrotron light, which is 0.8 for the MSB beamline, β is the incidence angle of the synchrotron light (10° for GI, 30° for NE, and 90° for NI), α is the tilt angle of the phenyl ring plane with respect to the surface plane.

S4. Low energy electron diffraction of cerium oxide films

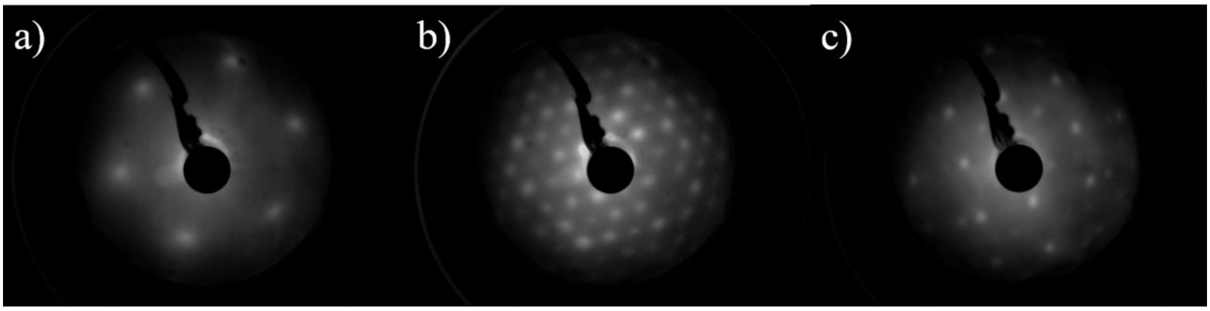

Fig. S3. LEED images of **a)** $CeO_2(111)/Cu(111)$, **b)** $Ce_2O_3(111)/Cu(111)$ and **c)** $Ce_6WO_{12}(100)/W(110)$ obtained at electron energies of 87, 60, and 134 eV, respectively.

S5. Ce 3d core level fitting

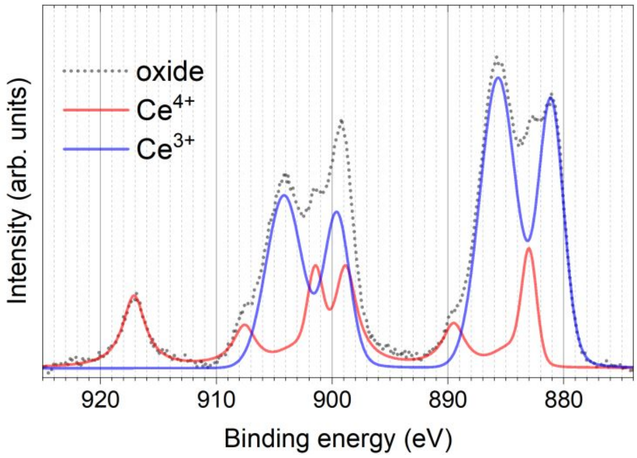

Fig. S4. Ce 3d core level spectra of $CeO_{1.7}$ oxide (dotted black line). Integrated Ce^{3+} (solid blue line) and Ce^{4+} (solid red line) components are shown for the clean $CeO_{1.7}$ oxide. The photon energy is 1486.6 eV.

S6. P 2p, C 1s, and O 1s core levels of the $CeO_{1.7}$ oxide film

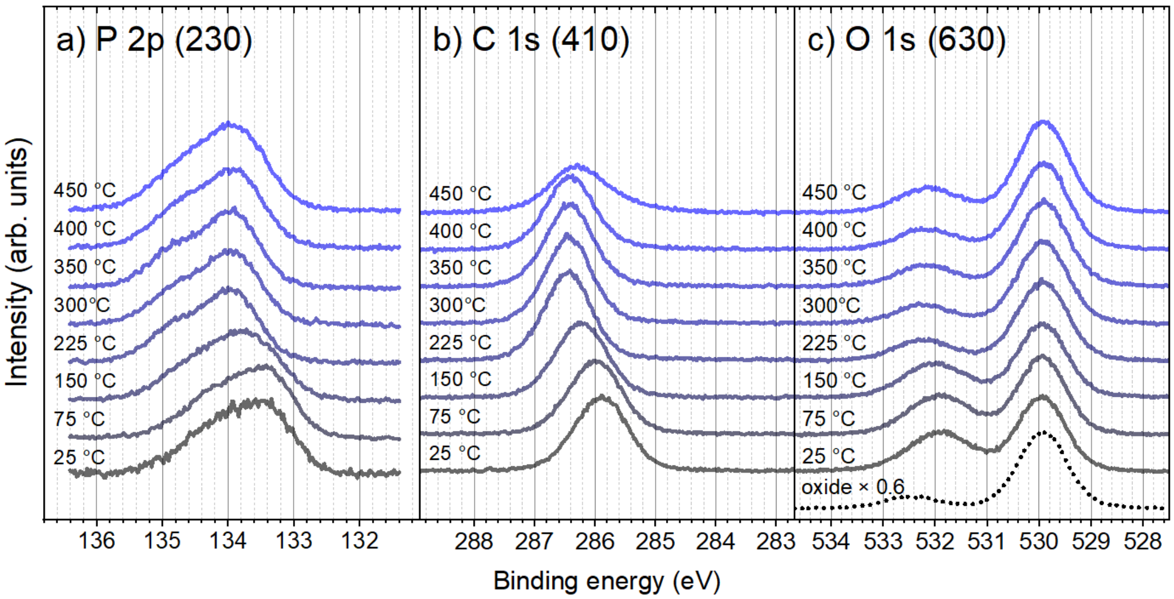

Fig. S5. a) P 2p, **b)** C 1s, and **c)** O 1s core level spectra of PPA on $CeO_{1.7}$ as-deposited and after subsequent annealing, measured with photon energy of 230, 410, and 630 eV, respectively. The dotted black O 1s spectrum was obtained before molecular deposition.

S7. C K-edge spectra of the $CeO_{1.7}$ oxide film

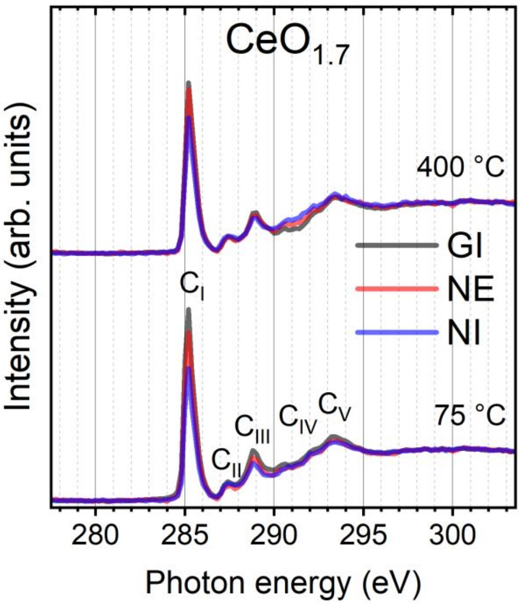

Fig. S6. C K-edge NEXAFS spectra of PPA on $CeO_{1.7}$ measured after annealing at 75 and 400 °C (blue, red, and black lines denote the NI, NE, and GI geometry of the substrate during the spectra acquisition, respectively).

S8. Estimation of the relative amount of cerium cations on the surface, the charge transferred from the PPA, and the oxygen lost from the surface

The relative amount of the two types of cerium cations on the surface can be estimated using the procedure described in Ref.[3], based on the proportionality of the RER value to the ratio of Ce^{3+} and Ce^{4+} centres on the surface with a coefficient of 5.5.

Estimation of the charge transfer from the PPA molecule to the CeO_2 surface:

0.07 ML PPA/CeO_2 system at 25 °C: The RER value of 0.05 gives a ratio of Ce^{3+} to Ce^{4+} cations on the surface of 0.05/5.5 or about 1/100, i.e., the formation of 1 Ce^{3+} centre per 100 Ce^{4+}. The PPA coverage of 0.07 ML allows a rough estimate that 1 e^- comes from an estimated (overestimated) 10 molecules, giving 0.1 e^- per PPA.

The oxygen loss from the surface can be estimated as follows:

1. 0.10 ML PPA/$CeO_{1.7}$ system at 25 °C: The RER value change of 1.1 gives a ratio of Ce^{3+} to Ce^{4+} cations on the surface[3] of 1.1/5.5 or about 20/100, i.e., 20 Ce^{3+} centres are formed per 100 Ce^{4+} or 10 oxygen atoms desorbed in the form of e.g. water. As a result,

the ratio of adsorbed PPA to expected desorbed H_2O molecules is about 1 to 1, considering the PPA coverage on $CeO_{1.7}$ of 0.10 ML.

2. 0.07 ML PPA/CeO_2 system after 450 °C: For the RER value of 0.9 the estimation gives 16 Ce^{3+} centres formed per 100 Ce^{4+}. Remembering that one oxygen atom desorbed from the surface creates 2 Ce^{3+} centres, 16 Ce^{3+} cations correspond to 8 desorbed O atoms. Using water desorption as an example, this corresponds to 8 molecules of H_2O. Since the coverage is 0.07 ML, i.e. 7 PPA molecules per 100 Ce atoms, we can conclude that there is approximately 1 desorbed H_2O per PPA molecule.
3. 0.39 ML PPA/CeO_2 system after 450 °C: For the RER value of 2.2, 38 Ce^{3+} centres are formed per 100 Ce^{4+}, i.e. approximately every third Ce cation is reduced, which is consistent with the saturation coverage and the mono- or bidentate adsorption geometry with one P–O bond deprotonated. In summary, 38 Ce^{3+} centres correspond to the 39 adsorbed PPA molecules and then to the desorption of 19 atoms of oxygen in carbonaceous species or 19 molecules of H_2O. We can therefore conclude that in this case there is approximately 0.5 desorbed H_2O per 1 PPA.

S9. P 2p, C 1s, and O 1s core levels, and C K-edge NEXAFS spectra of different PPA adlayers on the CeO_2 oxide films

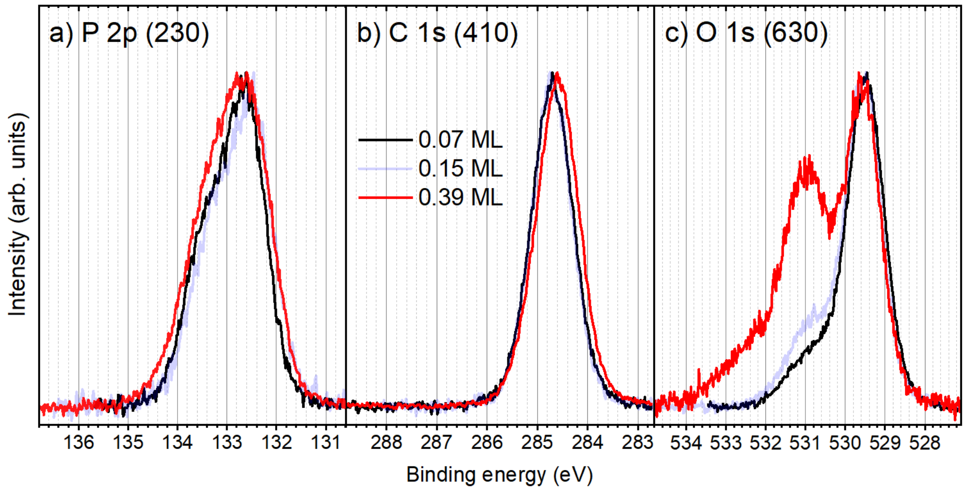

Fig. S7. a) P 2p, **b)** C 1s, and **c)** O 1s core level spectra of as-deposited PPA adlayers with coverage of 0.07, (black) 0.15, (light violet) and 0.39 ML (red) on CeO_2, measured with photon energy of 230, 410, and 630 eV, respectively.

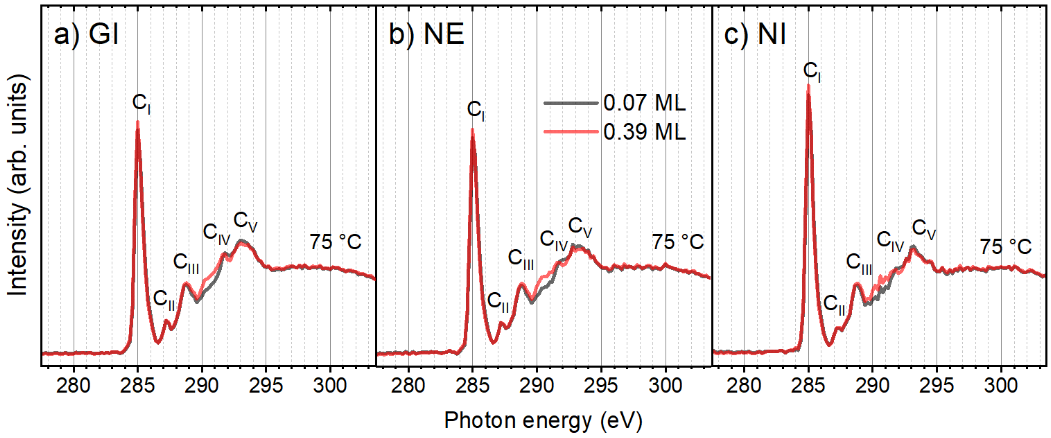

Fig. S8. C K-edge NEXAFS spectra of 0.07 (black) and 0.39 ML (red) PPA adlayers on CeO_2 after annealing at 75 °C, acquired in **a)** GI, **b)** NE, and **c)** NI geometries.

References

(1) Lytken, O.; Wechsler, D.; Steinrück, H.-P. Removing Photoemission Features from Auger-Yield NEXAFS Spectra. *J. Electron Spectros. Relat. Phenomena* **2017**, *218*, 35–39. https://doi.org/https://doi.org/10.1016/j.elspec.2017.05.012.

(2) Joachim Stöhr. *NEXAFS Spectroscopy*; Springer, 1996.

(3) Lykhach, Y.; Kozlov, S. M.; Skala, T.; Tovt, A.; Stetsovych, V.; Tsud, N.; Dvorak, F.; Johanek, V.; Neitzel, A.; Myslivecek, J.; Fabris, S.; Matolin, V.; Neyman, K. M.; Libuda, J. Counting Electrons on Supported Nanoparticles. *Nat Mater* **2016**, *15* (3), 284–288.
